# Feather-inspired janus interfaces with spatiotemporal ionic programming for diabetic wound infection control and regenerative healing

**DOI:** 10.1016/j.mtbio.2025.102506

**Published:** 2025-11-01

**Authors:** Chaoyang Huang, Lianglong Chen, Huihui Zhang, Bo Liu, Hai Zhou, Yanqi Chen, Xian Li, Xiaoyang Liu, Limin Zhao, Xue Wang, Min Wu, Shuaijie Li, Dan Yi, Chunyu Liu, Haobo Pan, Lei Yang

**Affiliations:** a Department of Burns, Nanfang Hospital, Southern Medical University, Jingxi Street, Baiyun District, Guangdong, 510515, PR China; b Institutes of Advanced Technology, Chinese Academy of Sciences, Shenzhen, Guangdong, 518055, Shenzhen, PR China; c Department of Burns and Plastic Surgery, Liuzhou Worker's Hospital, Fourth Affiliated Hospital of Guangxi Medical University, Liuzhou, Guangxi, 545000, PR China; d Yunfu People's Hospital, Central Laboratory of Yunfu People's Hospital, Yunfu City, 527399, PR China; e Geriatric Medicine Department, Shenzhen Longhua District Central Hospital, Shenzhen, 518000, PR China; f Shenzhen Healthemes Biotechnology Co., Ltd, Shenzhen, 518102, PR China

**Keywords:** Infected diabetic wounds, Feather-inspired topologies, Asymmetric janus interfaces, Antibacterial self-cleaning surfaces, Borosilicate bioactive glass, Spatiotemporal ionic programming

## Abstract

Recalcitrant wounds, such as diabetic foot ulcers (DFUs), pose a significant challenge to current therapeutic options due to persistent exudate, high infection risk, and a complex pathological microenvironment. This necessitates a novel dressing that can simultaneously provide both a physical barrier and active biological intervention. Inspired by the feathers of waterfowl, we developed a hierarchically engineered Janus dressing (PPT@PG-BG) via electrospinning, which integrates a hydrophobic antibacterial outer layer with a hydrophilic inner layer for the spatiotemporally controlled release of therapeutic ions (Mg^2+^, Zn^2+^, and Ce^3+^). This “external defense and internal regulation” strategy effectively re-establishes immune-angiogenic homeostasis by activating the p-ERK/Nrf2 antioxidant pathway, promoting M2 macrophage polarization, and enhancing neovascularization, ultimately achieving accelerated wound healing (97.85 % closure) in a diabetic infected wound model. In summary, this Janus dressing, which combines a simple fabrication process with integrated physical protection and multi-ion synergistic biotherapy, provides an effective strategy with significant clinical translation potential for treating complex, hard-to-heal wounds and promises to substantially improve patient outcomes.

## Highlights

• A feather-inspired Janus fibrous construct (PPT@PG-BG) is engineered by integrating biomimetic topographical architecture with programmable ionic microenvironment modulation, enabling spatiotemporally coordinated intervention for chronic diabetic wound repair.
• The superhydrophobic outer layer functions as a multifunctional barrier, effectively inhibiting microbial adhesion and biofilm development through synergistic physical shielding, wettability control, and sustained antibacterial activity.
• The bioactive inner layer dynamically reconstructs the pathological wound microenvironment by releasing therapeutic ions, thereby mitigating oxidative stress, modulating inflammation, and promoting angiogenesis to accelerate tissue regeneration.

## Graphical abstract

**Figure:**
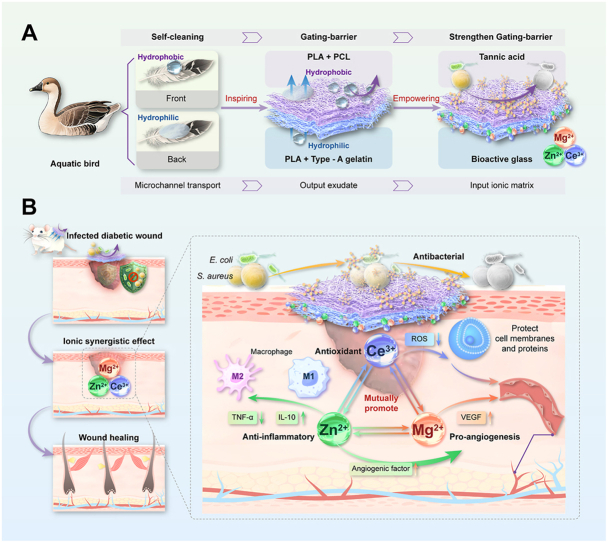

## Teaser

Significance: This bilayer “external protection-internal regulation” dressing combines an antimicrobial barrier with spatiotemporal ion programming to prevent infection and promote diabetic wound healing, offering a novel and promising solution for complex wound repair.

## 1 Introduction

**Scheme 1:**
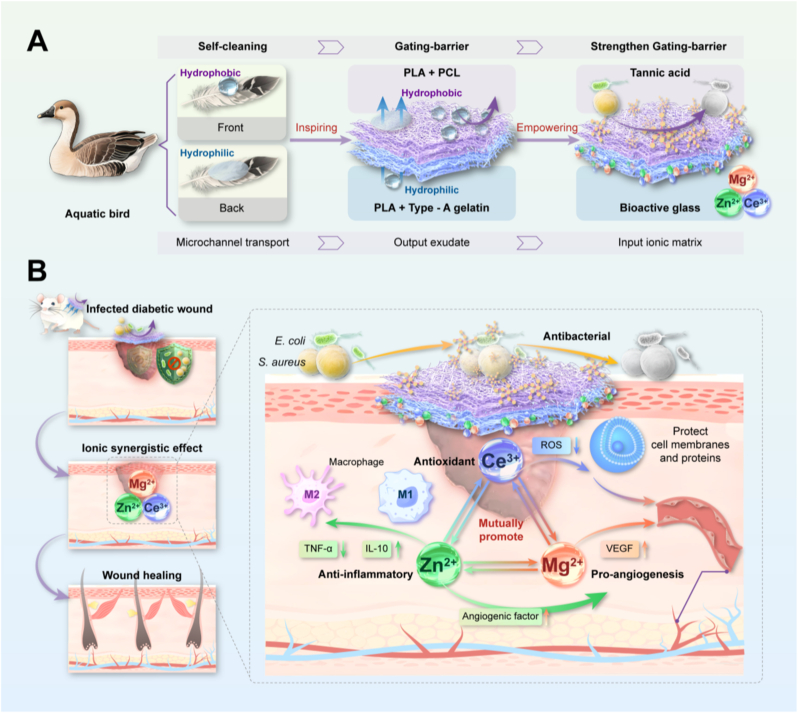
(A). The bioinspired fabrication process of the Janus fibrous pad mimics the hierarchical and asymmetric wettability of waterfowl feathers, with a hydrophobic PPT-modified outer layer and a hydrophilic PG-BG-infused inner layer. (B). The external layer serves as a waterproof, antibacterial barrier preventing exogenous infection. In contrast, the inner layer orchestrates a spatiotemporal ionic microenvironment that synergistically regulates inflammation, promotes angiogenesis, and accelerates chronic diabetic wound healing.

Diabetic foot ulcers (DFUs) pose a substantial global health threat, affecting approximately 15 %–34 % of individuals with diabetes. They account for around 84 % of limb amputations, significantly increasing the risk of mortality among affected patients [1,2]. DFU wounds are continuously exposed to a hyperosmotic microenvironment characterized by persistent inflammatory responses, excessive exudate, and high glucose concentrations [3]. These conditions lead to dysfunction in critical repair cells, including macrophages, endothelial cells, fibroblasts, and epithelial cells. This cascade of changes impairs angiogenesis and delays skin barrier repair, establishing a pathological cycle characterized by a “hyperosmotic-hypoxic-chronic inflammation” feedback loop that exacerbates the situation [4]. As a result, these factors markedly hinder the wound-healing process. Moreover, the hyperosmotic environment facilitates the formation of biofilm structures by common infectious pathogens, such as *Staphylococcus aureus*. This decreases the efficacy of local antibiotic penetration and further complicates efforts to control the infection [5,6] (see Scheme 1).

In the management of diabetic foot ulcers (DFUs), precise control of exudate, dynamic regulation of the wound microenvironment, and mitigation of infection risk jointly determine the efficiency and quality of healing [3,7]. Conventional hydrophilic dressings can transiently maintain a “moist” milieu and absorb exudate, but their action is largely passive: retained exudate perturbs local composition and osmotic balance, suppressing fibroblast and keratinocyte proliferation and migration, while also fostering microbial colonization; moreover, frequent dressing changes can mechanically damage nascent tissue and further delay repair [8,9]. These limitations underscore the need for next-generation dressings and management strategies that actively address exudate burden and microenvironmental dysregulation to effectively accelerate wound healing.

The new generation of Janus-interface dressings, characterized by their signature hydrophilic/hydrophobic bilayer structure, has offered a preliminary solution to the challenge of wound exudate management [10,11]. The core design principle is to absorb exudate with the hydrophilic inner layer while the hydrophobic outer layer prevents external contamination and bacterial infiltration, thereby achieving a basic physical compartmentalization of the wound microenvironment [12]. However, existing research has largely focused on optimizing this fundamental model—for instance, by constructing microchannels or adjusting fiber morphology to enhance unidirectional moisture-wicking efficiency [13]. These designs are inherently passive: their protective function is significantly compromised once exudate absorption reaches saturation or when faced with high-concentration bacterial colonization [14]. More importantly, they generally lack the active biological functions required to modulate complex pathological processes, such as persistent inflammation or impaired angiogenesis. This has severely limited their application in recalcitrant wounds, such as diabetic foot ulcers.

To overcome these limitations, we sought inspiration from nature. The micro- and nanostructure of waterfowl feathers demonstrates a perfect survival strategy of “external defense and internal regulation” [15]. Nanoscale grooves on the surface of the feather barbules, in synergy with a hydrophobic wax layer, physically block the adhesion of contaminants and pathogens. Simultaneously, the unique porous internal structure actively regulates temperature and humidity to provide a stable microenvironment for the feather follicle cells [[16], [17], [18]]. This strategy of seamlessly integrating a highly efficient physical barrier with active biological regulation offers an ideal blueprint for the design of the next generation of smart Janus dressings.

In recent years, the multifaceted roles of trace micronutrients in wound repair have become increasingly evident. Zinc (Zn) and copper (Cu) facilitate the scavenging of free radicals by activating superoxide dismutase (SOD) and associated redox enzyme systems [19]. Iron (Fe), through oxygen transport and the upregulation of vascular endothelial growth factor (VEGF), promotes angiogenesis, whereas calcium (Ca) and magnesium (Mg) activate TGF-β1 and the GSK3-CREB signaling pathway, thereby regulating collagen synthesis and the expression of keratinization-related genes such as COL1A1 and HAS2 [[20], [21], [22]]. To fully harness the synergistic reparative effects of these metal ions, precise spatiotemporal release within the wound milieu is essential to prevent antagonistic interactions such as Cu/Zn competition and to maintain redox homeostasis [23]. Borosilicate bioactive glass (BG), by its excellent biocompatibility and controllable release of multivalent ions, including BO_3_^3−^, SiO_4_^4−^, and Ca^2+^, has emerged as an ideal platform for establishing biomimetic microenvironments of bioactive ions [24,25].

This study deeply integrates a feather-inspired biomimetic interface design with the ion-programming mechanism of bioactive glass to construct a novel Janus wound dressing (PPT@PG-BG). Through an integrated “external defense and internal regulation” strategy, this dressing aims to achieve multifunctional synergistic therapy. Its outer layer, composed of hydrophobic polymers (PCL/PLA) and plant-derived tannic acid, forms a dual “physical-chemical” barrier that effectively prevents bacterial adhesion and biofilm formation. Its inner layer, which mimics the extracellular matrix, is loaded with magnesium-, zinc-, and cerium-doped bioactive glass nanoparticles to enable the spatiotemporally controlled release of multiple therapeutic ions. These ions act synergistically to suppress oxidative stress, modulate the inflammatory response towards pro-regenerative M2 macrophage polarization, and enhance neovascularization. The asymmetric wettability of the dressing facilitates unidirectional drainage of wound exudate. This feature, combined with the continuous regulation of the pathological microenvironment (e.g., hyperosmolarity, hypoxia) via ion programming, ultimately leads to significantly accelerated healing of diabetic foot ulcers. This work provides a new scientific basis and translational potential for the multi-dimensional regulation and clinical treatment of complex, infected wounds.

## 2 Materials and methods

### 2.1 Material

Type A gelatin, polycaprolactone (PCL), polylactic acid (PLA), tannic acid (TA), cetyltrimethylammonium bromide (CTAB), tetraethyl orthosilicate (TEOS), tributyl borate (TBB), triethyl phosphate (TEP), calcium nitrate tetrahydrate (CN), zinc nitrate hexahydrate (ZN), cerium (III) nitrate hexahydrate (CeN), magnesium nitrate hexahydrate (MN), KH-570 (SCRC), and ammonia solution (30 %, SCRC) were all purchased from Aladdin (China). The immortalized HUVEC cell line and HaCaT cells were obtained from Bioher (Shanghai) Biotechnology Co., Ltd. L929 murine fibroblasts and RAW 264.7 macrophages were sourced from the Clinical and Medical Laboratory Center, Nanfang Hospital, Southern Medical University. *Escherichia coli* and *Staphylococcus aureus* strains were obtained from the Guangzhou Institute of Microbiology. For animal experiments, Kunming mice (10 weeks old, 25 ± 3 g) were provided by the Animal Research Center of Southern Medical University.

### 2.2 Preparation of borate bioactive glass

The borosilicate-based bioactive glass (SiO_2_-B_2_O_3_-P_2_O_5_-CaO-MgO-ZnO-CeO_2_) was synthesized using a sol-gel method previously established by our group [26]. Briefly, 0.7 g of CTAB and 20 mL of ethyl acetate were added to 40 mL of deionized water, followed by stirring at a constant temperature of 30 °C. After 30 min, 0.7 mL of ammonia solution was slowly introduced. Subsequently, 2.88 mL of TEOS, 0.64 mL of TBB, 0.32 mL of TEP, 5.87 g of calcium nitrate tetrahydrate, 1.96 g of magnesium nitrate hexahydrate, 0.285 g of zinc nitrate hexahydrate, and 0.41 g of cerium (III) nitrate hexahydrate were sequentially added at 30-min intervals. Following 4 h of reaction, the mixture was centrifuged to collect the precipitate, which was then washed three times each with anhydrous ethanol and deionized water. The resulting product was freeze-dried at −80 °C for 48 h to obtain the glass precursor. Finally, the precursor was calcined at 500 °C for 4 h to yield the borosilicate bioactive glass.

### 2.3 Preparation of electrospinning

#### 2.3.1 The outer layer was prepared by electrospinning

Polycaprolactone (PCL, 0.5 g) and polylactic acid (PLA, 0.5 g) were weighed at a mass ratio of 1:1 and dissolved in 10 mL of hexafluoroisopropanol (HFIP) under continuous stirring overnight to obtain a homogeneous 10 wt% PCL/PLA electrospinning solution. Various amounts of tannic acid (TA) particles (0.1 %–1.0 % wt/v) were added to the PCL/PLA solution and dispersed by ultrasonication for 30 min to ensure complete dispersion of the TA particles. The resulting spinning solution was loaded into a 10 mL screw-cap syringe, fitted with a 20 G needle, and electrospun using an electrospinning apparatus at a flow rate of 1.5 mL/h under a high voltage of 15–18 kV, with fibers collected on a rotating drum (120 rpm) positioned 15 cm from the needle tip. Fibrous membranes with different TA contents were prepared by incorporating 0.1 %, 0.2 %, 0.5 %, and 1 % (wt/v) TA into the PCL/PLA solution and are denoted as 0.1 %-TA, 0.2 %-TA, 0.5 %-TA, and 1 %-TA, respectively. The fibrous membrane fabricated from PCL/PLA solution without TA particles was the control group (PP). Subsequent surface hydrophobicity, air permeability, and cellular compatibility were evaluated (see Fig. S2–S3). The fibrous membrane modified with 1.0 % TA exhibited the most favorable performance and was selected as the outer substrate, hereafter referred to as PPT.

#### 2.3.2 The inner layer electrospinning preparation

Gelatin (0.96 g) and polylactic acid (PLA, 0.24 g) were weighed at a mass ratio of 4:1 and dissolved in 10 mL of hexafluoroisopropanol (HFIP) under constant stirring overnight to yield a 12 wt % gelatin/PLA electrospinning solution. Bioactive glass (BG) nanoparticles at various concentrations (0.5 %–1.5 % wt/v) were then added to the gelatin/PLA solution and dispersed by ultrasonication for 30 min to achieve complete nanoparticle dispersion. The resulting spinning solution was loaded into a 10 mL screw-cap syringe fitted with a 20 G needle and electrospun at a rate of 2 mL/h under a high voltage of 15–18 kV directly onto the previously prepared PLA-PCL-TA fibrous membrane (PPT). The resulting composite membranes with varying BG contents were designated PPT@PG (without BG), PPT@PG-0.5 %BG, PPT@PG-1.0 %BG, and PPT@PG-1.5 %BG, respectively.

### 2.4 Characterization of materials

The electrospun fibrous membranes ' elemental distribution and surface morphology was characterized by scanning electron microscopy (SEM, Regulus 8100/8200, Hitachi, Japan) and transmission electron microscopy. Fiber diameters were quantitatively analyzed using ImageJ software. The chemical structures of BG nanoparticles and nanofiber membranes were confirmed by Fourier-transform infrared spectroscopy (FTIR). Spectra ranging from 4000 cm^−1^ to 400 cm^−1^ were acquired using a Nicolet iS50 F T-IR spectrometer (Thermo, USA). For X-ray diffraction (XRD) analysis, a SmartLab SE diffractometer (Rigaku, Japan) was used to detect the crystalline phases of samples over a 2θ range of 10°–80°. Hydrophilicity and hydrophobicity were assessed using a contact angle analyzer. The surface wettability of both the inner and outer electrospun layers was evaluated by depositing a five μL droplet of deionized water onto the sample surface and recording the process until the droplet vanished; images were extracted from these videos to calculate the contact angle (n = 3). For ion release analysis, samples were incubated in 0.9 % phosphate-buffered saline (PBS) at 37 °C, with supernatants collected at predetermined time points and replaced with an equal volume of fresh PBS. The concentrations of Mg^2+^, Zn^2+^, and Ce^3+^ were determined by inductively coupled plasma atomic emission spectrometry (ICP-AES, Prodigy Plus, Leeman, USA) (n = 3 per group). To evaluate the water vapor transmission rate (WVTR), samples were cut into squares measuring 10 × 10 × 2 mm. Each sample was placed over the opening of a 10 mL glass vial containing 5 mL of saline. After recording the initial weight of the vial, the assembly was incubated at 37 °C in a drying oven with 50 % humidity. The vial was weighed again after 24 h. WVTR was calculated using the following formula: WVTR = (W_0_ – W_1_)/(S × 24), where W_0_ is the initial weight, W_1_ is the weight after 24 h, and S is the area of the vial opening.

### 2.5 Biocompatibility testing

CCK-8 Assay: Electrospun samples were incubated in a complete culture medium for 24, 48, and 72 h. The extracts collected at each time point were filtered through a sterile filter to obtain the nanofibrous materials' 24-h, 48-h, and 72-h eluates. L929 cells were seeded into 96-well plates, and, after attachment, the culture medium was replaced with the different time-point extracts; the control group received fresh basal medium. On days 1 and 2 post-extract exposure, the extracts were removed and replaced with CCK-8 working solution, followed by incubation in the dark for 1 h. Absorbance was measured at 450 nm using a microplate reader to assess cytotoxicity. Live/Dead Cell Staining: HUVECs were seeded into 96-well plates and, after adherence, exposed to 72-h eluates; the control group was maintained in a complete culture medium. Cells were incubated in the extracts for 24, 48, and 72 h, washed with sterile PBS, and stained with AM/PI reagents in the dark. Fluorescence microscopy was used to evaluate cell viability, and live/dead cell fluorescence intensity was quantified using ImageJ software. Phalloidin Staining: HaCaT cells were seeded into 24-well plates with extract-containing medium and cultured for 24 h. Cells were then stained with DAPI/phalloidin fluorescent dyes to visualize the morphology and distribution of the cytoskeleton (F-actin). Hemolysis Assay: Two milliliters of fresh anticoagulated pig blood were mixed with 8 mL of PBS and centrifuged at 3000 rpm for 10 min. The resulting red blood cells were further diluted to a 2 % suspension. Various equal-mass inner nanofibrous membranes were pre-warmed in saline at 37 °C for 30 min, after which the diluted erythrocyte suspension was added to the samples. Following incubation at 37 °C for 1 h, the mixtures were centrifuged at 3000 rpm to collect the supernatant. The absorbance of the supernatant was measured at 540 nm using a microplate reader (n = 3 per group). Triton X-100 and saline were used as positive and negative controls, respectively. The hemolysis rate was calculated as follows: Hemolysis rate (%) = [(OD sample – OD negative control)/(OD positive control – OD_negative control)] × 100 %.

Scratch Wound Assay: Cells were seeded in 12-well plates and, after attachment, subjected to serum-free high-glucose medium for 24 h to induce starvation. A straight scratch was created using a 200 μL pipette tip; cells were rinsed 2–3 times with PBS. The inner electrospun membrane was placed in a Transwell insert and transferred into the wells. Cell migration was monitored at indicated time points using microscopy to assess wound closure.

### 2.6 Antibacterial related experiments

The antibacterial activity of the fibrous nanomembrane materials was evaluated by plate counting, bacterial live/dead staining, and morphological analysis. *Escherichia coli (E. coli)* and *Staphylococcus aureus (S. aureus)* were cultured to the logarithmic growth phase and co-incubated with sterilized samples. Distinct protocols were performed for subsequent analyses. Plate Counting: After co-incubation, bacterial suspensions were collected; untreated bacterial suspensions served as the control group. Samples were serially diluted, and 100 μL of each suspension was evenly spread onto agar plates. Antibacterial efficacy was quantified by comparing the number of colonies in experimental and control groups. The antibacterial rate was calculated as follows: Antibacterial rate (%) = (Number of colonies in control – Number of colonies in the experimental group)/Number of colonies in control × 100 %.

Bacterial Live/Dead Staining: Treated bacterial suspensions were stained according to the manufacturer's instructions for the live/dead bacterial viability kit. A 10 μL aliquot of stained suspension was dropped onto a glass slide and observed under a fluorescence microscope. The proportion of live bacteria was calculated as: Viability (%) = Green fluorescence intensity/(Green fluorescence intensity + Red fluorescence intensity) × 100 %.Morphological Analysis: Bacterial suspensions co-cultured with the materials were fixed with paraformaldehyde. Dehydration was performed using a gradient series of ethanol concentrations, followed by gold sputter-coating. SEM then visualized bacterial morphology.

### 2.7 Ion synergy correlation experiment

#### 2.7.1 Angiogenesis related experiments

The Matrigel matrix was thawed at 4 °C, and all subsequent procedures were conducted on ice. 100 μL of Matrigel was added to each well of a 24-well plate and allowed to solidify in a cell incubator. Once the Matrigel had gelled, cells were seeded onto the substrate. The seeding density of HUVECs on Matrigel was 2 × 10^4^ cells per well. The modified Transwell inserts with experimental materials were positioned above each well, and the plates were incubated for 20 h. The formation of capillary-like tube structures was subsequently observed under a microscope, and quantitative analysis was performed using ImageJ software. Using the Angiogenesis Analyzer plugin for ImageJ software, we quantified three randomly selected fields of view for each sample.

For immunofluorescence staining, cells were blocked for 30 min and then incubated overnight at 4 °C with a primary antibody against VEGF. Following incubation with a suitable secondary antibody, cell nuclei were counterstained with DAPI. Images were captured using an inverted optical microscope, and quantitative analysis of the results was performed with ImageJ software.

#### 2.7.2 Regulation correlation experiment

RAW 264.7 macrophages were seeded in 24-well plates and stimulated with LPS (200 ng/mL) for 24 h. Subsequently, cells were treated with PBS, PPT@PG extract, or PPT@PG-BG extract for 24 h. The expression of inflammatory (TNF-α) and anti-inflammatory (IL-10) cytokines was assessed by immunofluorescence staining and observed under a fluorescence microscope. To evaluate the influence of the materials on macrophage polarization, cells were first exposed to M1 induction medium (RPMI-1640 + 10 % FBS + LPS) or M2 induction medium (RPMI-1640 + 10 % FBS + IL-4) for 24 h. This was followed by treatment with PBS, EF extract, or EF-BG extract for 24 h. The favorable expression rates of INOS and CD163 were subsequently determined. The positive control group was designated as PCTRL, and the negative control group as NCTRL.

#### 2.7.3 Antioxidant correlation experiment

The antioxidant capacity of the samples was evaluated using the DPPH assay. A 0.1 mM DPPH solution was prepared, and the test samples were mixed with the DPPH solution, followed by incubation in the dark for 30 min. The absorbance was then measured at 517 nm. The scavenging rate was calculated as follows: Scavenging rate (%) = (A_0_ -A_1_)/A_0_ × 100 %, where A_0_ is the absorbance of the control and A_1_ is the absorbance of the sample. In addition, intracellular reactive oxygen species (ROS) levels were measured using the Reactive Oxygen Species Assay Kit (Beyotime), following the manufacturer's instructions.

### 2.8 In vivo experiments

Male Kunming mice (20–25 g) were rendered diabetic through a combination of streptozotocin (STZ, Sigma-Aldrich) injection and a high-sugar, high-fat diet. Mice were fed this diet for four weeks, after which they received intraperitoneal injections of STZ (50 mg/kg) for five consecutive days following a fasting period. Blood glucose was monitored daily; mice were deemed diabetic when fasting blood glucose levels consistently exceeded 16.7 mmol/L. Anesthesia was induced with 1 % pentobarbital sodium (50 mg/kg). A circular punch created two full-thickness excisional wounds (diameter: 12 mm) on the dorsal skin. Mice were randomly assigned to four groups (n = 3): Control, PP@PG, PPT@PG, and PPT@PG-BG. To establish the infected full-thickness skin defect model, a bacterial suspension of *Staphylococcus aureus* (ATCC 25923) was prepared. Briefly, immediately after the full-thickness excisional wound was created, 100 μL of the bacterial suspension, containing a total of 1 × 10^6^ CFUs of *S. aureus*, was topically applied to the surface of each wound. The wounds were then left exposed for 20 min before covering with the dressing to allow for bacterial adhesion. Fibrous membranes corresponding to each group were applied to the wound surface and secured with gauze dressings. Wound healing was observed and recorded on postoperative days 0, 3, 7, and 14. At each time point, tissue samples (collected within 0.5 cm of the wound edge) were fixed in 4 % paraformaldehyde, paraffin-embedded, sectioned, and subjected to hematoxylin and eosin (H&E) staining, Masson's trichrome staining, and immunohistochemical staining for CD31, p-ERK, TNF-α, and IL-10 to assess re-epithelialization, collagen deposition, angiogenesis, and inflammation. For immunofluorescence analysis, tissues were blocked with 5 % bovine serum albumin for 2 h at room temperature and incubated overnight at 4 °C with primary antibodies against VEGF, iNOS, CD206, and Nrf-2. Following this, sections were incubated with secondary antibodies at 37 °C for 1 h. Nuclei were counterstained with DAPI for 5 min at room temperature. Fluorescence images were acquired with a fluorescence microscope and quantified using ImageJ software. To elucidate the effects of PPT@PG-BG on tissue regeneration and repair, RNA sequencing (RNA-seq) was performed at the tissue level. RNA was extracted from wound tissues on postoperative day 7 using Trizol reagent. RNA concentration and quality were assessed using a Nanodrop ND-1000 spectrophotometer (Gene Company, USA) and a 2100 Bioanalyzer (Agilent). Purified RNA samples were subjected to library preparation and sequencing on the Illumina platform (USA).

### 2.9 Statistical analysis

Statistical analyses were performed using GraphPad Prism 10. Significant differences among groups were assessed by one-way analysis of variance (one-way ANOVA). Data are presented as mean ± standard deviation (SD). Statistical significance was defined as ∗*P* < 0.05, ∗∗*P* < 0.01, ∗∗∗*P* < 0.001, and ∗∗∗∗*P* < 0.0001.

## 3 Results and discussion

### 3.1 A biomimetic janus nanofiber membrane with asymmetric wettability was successfully fabricated

**Fig. 1:**
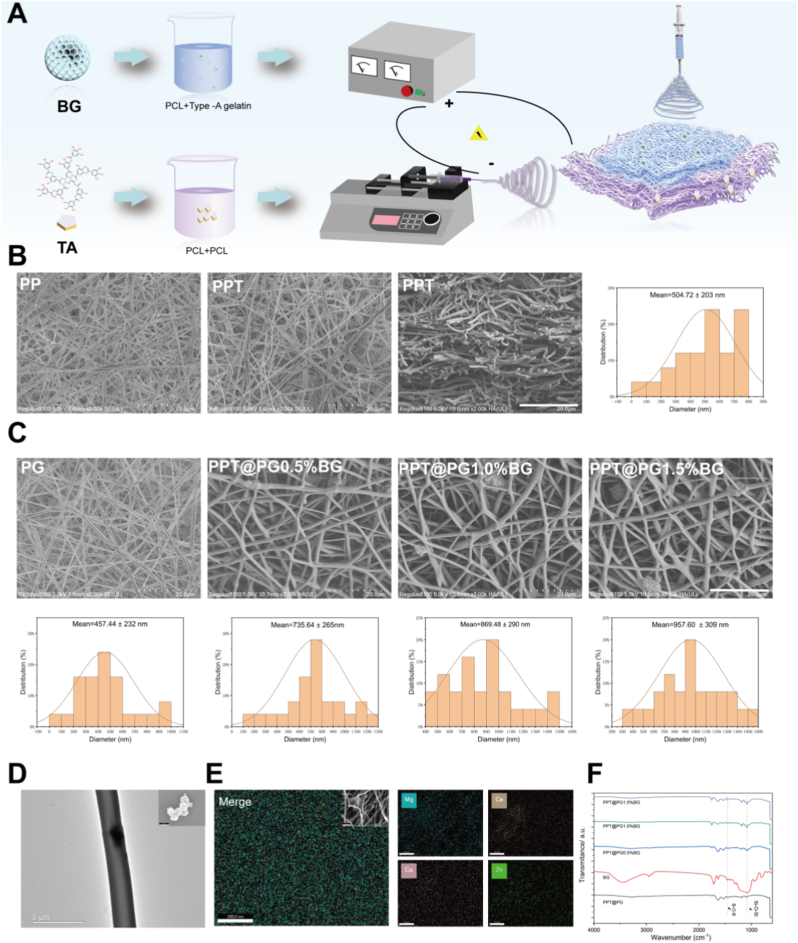
Construction and morphological characterization of PPT@PG-x%BG Janus nanofibrous membranes: (A) Schematic illustration of the material fabrication process, highlighting the formation of the hydrophobic outer PPT layer and the hydrophilic PG nanofibrous membrane loaded with BG as the inner layer; (B) SEM image of the outer PLA-PCL@TA (PPT) electrospun membrane, illustrating its porous and anisotropic architecture; (C) Structural morphology of the inner PLA-gelatin electrospun membranes and their composites with varying BG contents, as shown by SEM images of PG, PPT@PG-0.5 %BG, PPT@PG-1.0 %BG, and PPT@PG-1.5 %BG; corresponding statistical analysis of nanofiber diameter distributions are presented below; (D) TEM image of a representative composite fiber, revealing BG particles embedded within the nanofiber matrix; (E) EDS elemental mapping of the composite fibrous membranes, confirming the presence of active elements including Mg, Zn, Ce, and Ca; (F) FT-IR spectra of PPT@PG-x%BG membranes. (PP denotes PCL-PLA; PPT denotes PCL-PLA@TA; PG denotes PLA-gelatin; and PG-BG denotes PLA-gelatin@BG.)

Inspired by the distinctive Janus interfacial architecture and asymmetric wettability of waterfowl feathers, this study has engineered a biomimetic nanofibrous Janus membrane with dual functional capabilities. This material is composed of two distinct functional layers. The outer barrier layer possesses hydrophobicity and antibacterial properties, effectively preventing the penetration of external liquids and the invasion of pathogenic microorganisms. The inner layer is endowed with microenvironment-responsive regulatory capability, enabling the sustained release of bioactive ions to emulate the physicochemical characteristics of the native extracellular matrix (ECM). This design allows for dynamic modulation of wound-site parameters, including moisture, pH, and cellular adhesion and migration, thus providing a favorable physiological milieu for tissue repair and regeneration. The Janus membrane is fabricated via electrospinning with a hydrophilic PLA-gelatin composite nanofibrous membrane (loaded with varying concentrations of borosilicate bioactive glass [BG] particles, X = 0.5 %, 1.0 %, and 1.5 % w/w) serving as the inner layer, deposited onto the surface of the hydrophobic PLA-PCL@TA (PPT) fibrous membrane to generate a directionally structured Janus interface. The preparation process comprises three principal steps: (a) synthesis of nanoscale borosilicate bioactive glass (BG, with a particle diameter of approximately 163.73 ± 19.21 nm, as shown in Fig. S1B); (b) formulation of PLA-PCL@TA and PLA-gelatin@BG electrospinning solutions; and (c) construction and sequential assembly of the two fibrous membranes via electrospinning (Fig. 1A). During the fabrication of the outer hydrophobic PPT membrane, the effects of varying tannic acid (TA) concentrations (0.1 %, 0.2 %, 0.5 %, and 1.0 % w/w) on the surface hydrophobicity, breathability, and cytocompatibility of the PLA-PCL membrane were systematically evaluated (see Figs. S2–S3). The results demonstrated that the PLA-PCL@TA fibrous membrane modified with 1.0 % TA exhibited optimal performance across all evaluated parameters and was thus selected as the outer substrate (hereafter abbreviated as PPT). On this basis, PLA-gelatin composite fibrous membranes loaded with different concentrations of BG were subsequently deposited onto the PPT layer, yielding a series of Janus membranes designated as PPT@PG (no BG), PPT@PG-0.5 %BG, PPT@PG-1.0 %BG, and PPT@PG-1.5 %BG. This allowed for a systematic exploration of the influence of BG content on the structural and functional regulatory capabilities of the resulting membranes.

To comprehensively analyze the microstructural morphology and chemical composition of the PPT@PG-x%BG Janus nanofibrous membranes, a systematic characterization was conducted using scanning electron microscopy (SEM), Fourier transform infrared spectroscopy (FT-IR), and transmission electron microscopy (TEM) coupled with energy-dispersive X-ray spectroscopy (EDS) mapping. SEM analysis revealed that the PPT layer (hydrophobic outer layer) consists of anisotropic, loosely porous fibers with an average diameter of 504.72 ± 203.7 nm (Fig. 1B), facilitating the formation of an effective barrier against liquids and microorganisms. FT-IR analysis further confirmed the successful incorporation of tannic acid (TA) into the PLA-PCL fibers (PP). Compared with the pure PLA-PCL (PP) control group, the PPT membrane exhibited a pronounced absorption band corresponding to the carboxyl C=O stretching vibration near 1700 cm^−1^, as well as a characteristic absorption peak for aromatic ring C=C skeletal vibrations at 1450 cm^−1^. These spectral changes indicate the effective incorporation of polyphenolic functional groups from TA onto the fibrous substrate (see Fig. S3D for details) [27]. For the PG-x%BG layer (hydrophilic inner layer), SEM observations (Fig. 1C) revealed that in the absence of BG incorporation, the resulting fibers exhibited a relatively uniform and smooth morphology, with an average diameter of 457.44 ± 232.7 nm. With increasing BG content (x = 0.5 %, 1.0 %, 1.5 % w/w), the fiber diameter increased markedly, rising from 735.64 nm to 957.60 nm. This enlargement is presumed to result primarily from incorporating inorganic BG particles into the polymer matrix during electrospinning. Further TEM analysis (Fig. 1D) revealed that BG particles were uniformly embedded within the nanofibers, while SEM images (Fig. 1C) also showed BG particles attached to the fiber surfaces. EDS elemental mapping (Fig. 1E) further confirmed that Mg, Zn, Ce, and Ca from the BG were successfully introduced into the fibrous membrane structure, demonstrating a uniform elemental distribution throughout the membrane. FT-IR data (Fig. 1F) showed pronounced changes in the absorption bands near 1450 cm^−1^ and 1100 cm^−1^ with increasing BG content, which are attributed to the skeletal vibrations of B-O-B (−1450 cm^−1^) and the symmetric stretching vibrations of Si-O-Si/B-O (−1100 cm^−1^), respectively [24,28]. These spectral features further confirm BG's effective incorporation and structural integration within the fibers. These multidimensional characterization results conclusively demonstrate the successful fabrication of the biomimetic PPT@PG-x%BG Janus nanofibrous membranes and highlight the coupling between their structure and functionality.

### 3.2 The PPT@PG-BG membrane exhibits excellent biocompatibility

**Fig. 2:**
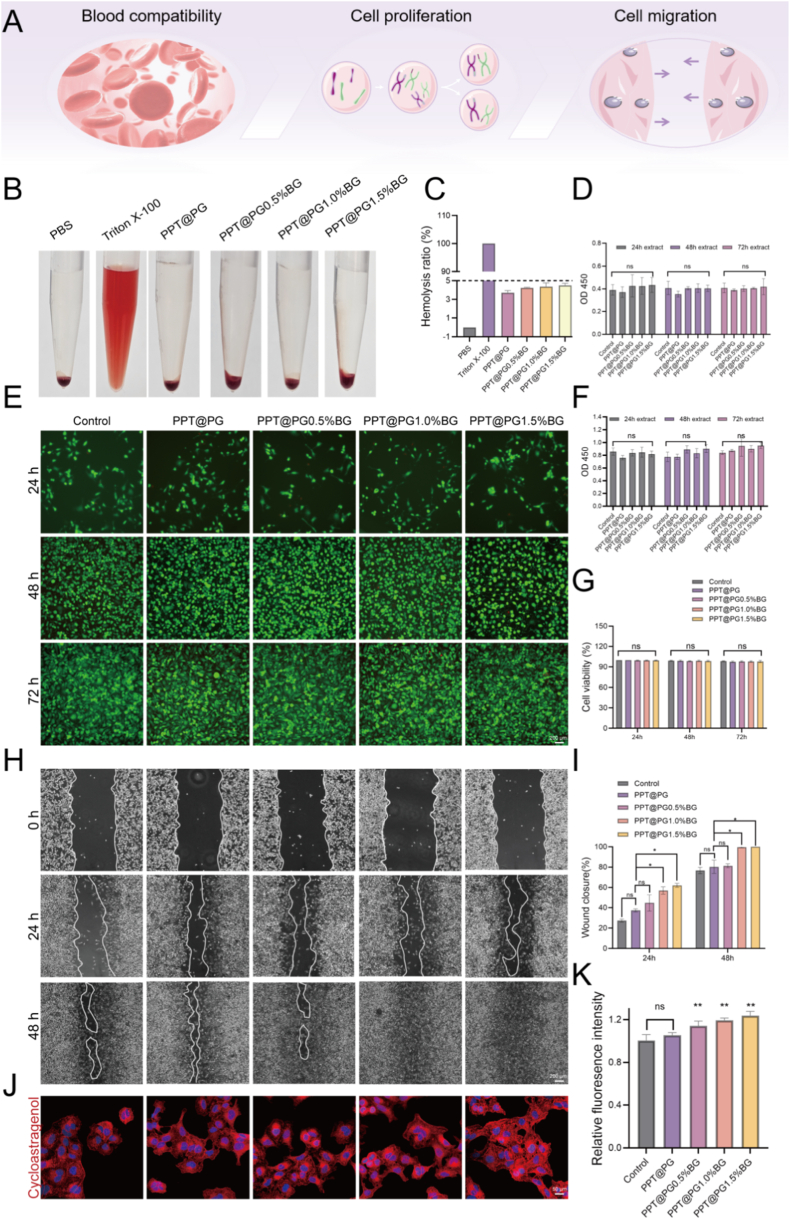
Biocompatibility evaluation and characterization of cellular behaviors: (A) Schematic illustration of the experimental procedures for assessing material biocompatibility; (B) macroscopic images of hemolysis rates for each PPT@PG-x%BG group; (C) quantitative analysis of hemolysis rates for different samples; (D, F) CCK-8 assay results showing cell viability of L929 cells after 24 h and 48 h co-culture with PPT@PG-x%BG sample extracts prepared at various time points; (E) Calcein-AM/PI fluorescence staining images of live and dead HUVECs after 72 h co-culture with PPT@PG-x%BG extracts; (G) quantitative analysis of fluorescence intensity from live/dead cell staining; (H) representative scratch wound healing images of L929 cells after 24 h and 48 h co-culture with different sample extracts; (I) quantitative analysis of cell migration distances in the scratch assay; (J) fluorescence images showing F-actin (phalloidin) staining of HaCaT cells after 72 h co-culture with PPT@PG-x%BG extracts; (K) quantitative analysis of F-actin fluorescence intensity. (∗*p* < 0.05, ∗∗*p* < 0.01, ns, no significant difference).

To evaluate the biosafety of the engineered PPT@PG-x%BG biomimetic nanofibrous membranes and their capacity to modulate cellular behaviors critical for tissue regeneration, we systematically conducted comprehensive assessments of biocompatibility and cell function. Robust biocompatibility is a fundamental prerequisite for the clinical translation of wound dressing materials. Hemocompatibility of the PPT@PG-x%BG fibrous membranes was first assessed via hemolysis assays. According to GB/T 16886.4–2003 Biological Evaluation, a hemolysis rate below 5 % reflects negligible hemolytic activity toward erythrocytes [29]. As shown in Fig. 2C, all tested groups-including PPT@PG, PPT@PG-0.5 %BG, PPT@PG-1.0 %BG, and PPT@PG-1.5 %BG-exhibited hemolysis rates well below this threshold, indicating that these materials do not elicit significant hemolytic responses upon contact with blood, thereby demonstrating excellent hemocompatibility. The cytocompatibility of the materials was further investigated using the CCK-8 assay. Given the sustained release of functional metal ions from the incorporated bioactive glass (BG), extracts of BG-loaded electrospun membranes (collected after 24, 48, and 72 h of incubation) were co-cultured with L929 cells for both 24 and 48 h. Subsequent measurement of cell viability (OD values; Fig. 2D–F) revealed no statistically significant differences (*p* > 0.05) between all BG-containing groups and the control group. This indicates that BG incorporation does not adversely influence cell proliferation, and that the materials overall possess good cytocompatibility. To further assess cellular health, Calcein-AM/PI dual staining was performed to distinguish live and dead cells. Following treatment with 72-h extracts, HUVECs demonstrated healthy, adherent growth (Fig. 2E). Moreover, after 72 h of co-culture, a marked increase in green fluorescence was observed across all groups, with no significant differences among them (Fig. 2G), further corroborating the low cytotoxicity and outstanding biosafety of the materials.

Cell migration ability is a critical indicator for evaluating the potential of wound dressing materials to promote tissue regeneration [30]. This study employed a scratch assay to simulate the wound-healing process and investigate the effects of varying BG concentrations on L929 cell migration. As shown in Fig. 2H, the PPT@PG-1.5 %BG group demonstrated nearly complete coverage of the scratch area by cells at 48 h, indicating significantly enhanced migratory capacity. Quantitative analysis further revealed that migration distances in the PPT@1.5 %BG group were markedly higher than those in the PPT@PG group and the blank control at 24 and 48 h, with statistically significant differences (*p* < 0.05, Fig. 2I).

Additionally, phalloidin staining was used to visualize the cytoskeletal architecture of HaCaT keratinocytes (Fig. 2J). The results showed that cells in the PPT@1.5 %BG group exhibited more spread morphologies, with well-defined F-actin microfilaments and more complete cytoskeletal reconstruction. Quantifying fluorescence intensity also revealed a significant increase in the PPT@1.5 %BG group compared to the control (*p* < 0.01, Fig. 2K). This phenomenon may be attributed to the sustained release of bioactive ions from BG, which regulates cytoskeletal organization and facilitates cellular adhesion and migration [31,32]. Taken together, the PPT@PG-x%BG electrospun membranes exhibited excellent hemocompatibility and cytocompatibility. At the same time, the 1.5 %BG-loaded group showed the most pronounced enhancement in cell migration and cytoskeletal remodeling, indicating its strong potential to promote wound healing. Therefore, the PPT@PG-1.5 %BG group was selected as the optimal formulation for subsequent studies and is hereafter referred to as PPT@PG-BG for in-depth mechanistic investigation and animal.

### 3.3 Physicochemical and transport properties of the PPT@PG-BG membrane were characterized

Imbalanced exudate management and retrograde ingress of external fluids are among the principal pathological drivers of chronic nonhealing in diabetic wounds [33]: the former sustains abnormal fluid accumulation, thereby suppressing cellular proliferation and migration and hindering tissue reconstruction; the latter conveys microorganisms into the wound against the barrier, disrupting the established physical interface and precipitating secondary infection. However, conventional single-mode hydrophilic or hydrophobic dressings are functionally constrained and cannot concurrently block contamination while stabilizing the wound microenvironment [34,35]. To overcome these limitations, we engineered an asymmetrically wettable Janus membrane (PPT@PG-BG) that implements a spatiotemporal division of labor—“external protection against contamination, internal modulation of the microenvironment”.

**Fig. 3:**
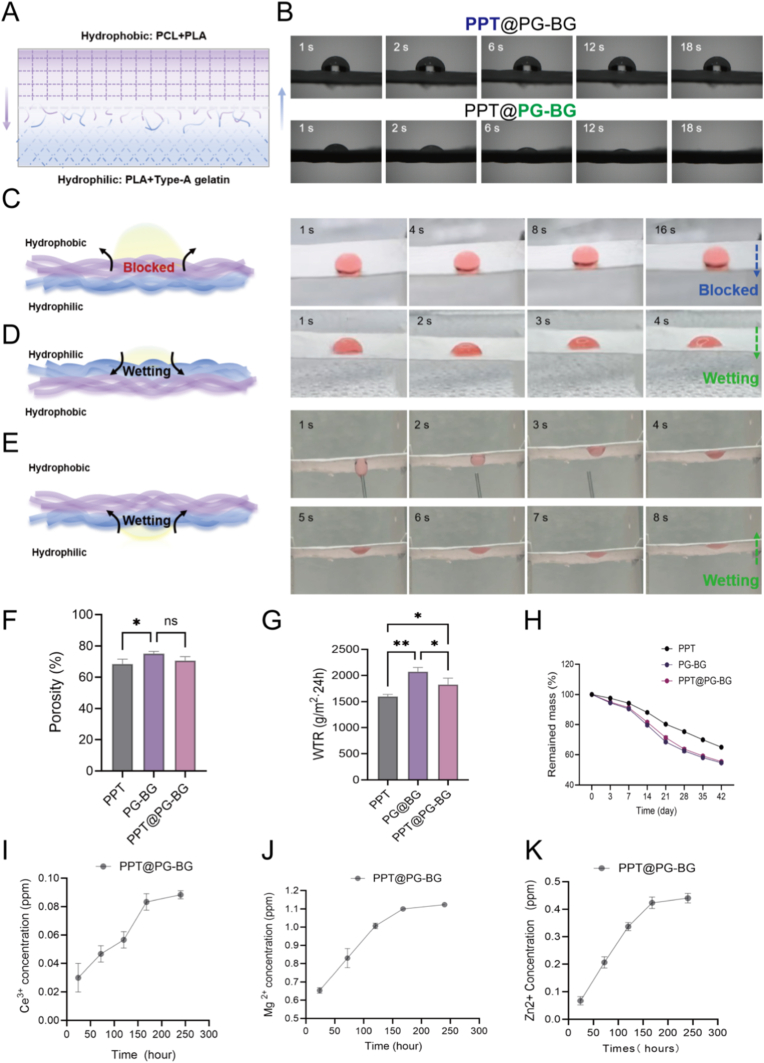
Physicochemical and transport properties of PPT@PG-BG. (A) Schematic of the bilayer electrospun Janus architecture: hydrophobic PPT outer layer and hydrophilic PG-BG inner layer.(B) Water contact angles of the hydrophobic and hydrophilic surfaces. (C–E) Conceptual diagrams and time-lapse images of droplet–membrane interactions under different orientations. (F) Porosity comparison of single-layer versus Janus membranes. (G) Water vapor transmission rate (WVTR). (H) In vitro degradation profiles. (I–K) Cumulative release profiles of Mg, Zn, and Ce ions from PPT@PG-BG.(∗p<; 0.05, ∗∗p < 0.01, ns, no significant difference).

First, structural and wettability assessments showed that the hydrophobic outer layer (PPT) exhibited a water contact angle of approximately 120°, with droplets remaining stably hemispherical, thereby forming a persistent barrier to liquid ingress; by contrast, the hydrophilic inner layer (PG-BG) reduced the contact angle to 50° within 1 s and fully absorbed the droplet within 12s, indicating rapid wetting and controlled uptake (Fig. 3B). Dynamic droplet imaging further revealed that, with the hydrophobic face oriented upward, droplets neither spread nor penetrate and remain stable; with the hydrophilic face upward, droplets disperse gradually and are progressively incorporated into the fibrous layer, while the hydrophobic layer effectively prevents through-thickness penetration (Fig. 3C–D). In a more clinically relevant configuration (hydrophilic layer facing the wound, hydrophobic layer outward), the hydrophilic layer “wicks against gravity,” drawing in exudate from below and enabling slow in-layer diffusion, thereby preventing sudden flooding and marginal maceration (Fig. 3E). This process maintains a “moist-but-non-accumulating” steady state and provides a stable conduit for the diffusion of drugs or bioactive ions [35]. Consistent with this robust fluid management, in vitro release tests demonstrated that PPT@PG-BG delivers Mg, Zn, and Ce ions in a slow and sustained manner, with characteristic sustained-release profiles (Fig. 3I–K), thereby conferring concerted antioxidant, anti-inflammatory, and proangiogenic effects that support extracellular matrix (ECM) reconstruction and tissue repair. Moreover, structure–property analyses confirmed that, relative to single-layer PPT, PPT@PG–BG exhibits higher porosity and a tuned water vapor transmission rate (WVTR) (Fig. 3F–G), ensuring that absorbed exudate is accommodated within the layer without breakthrough. In parallel, the glass phase promotes dressing degradation while continuously releasing active ions (Fig. 3H). Collectively, by coupling a durable outer barrier with controlled inner absorption and sustained release, PPT@PG-BG disrupts the exudate imbalance and external backflow that precipitate infection, stabilizes the wound microenvironment, and demonstrates clear translational potential for chronic, nonhealing diabetic wounds.

### 3.4 The PPT@PG-BG membrane exhibits potent antibacterial activity against both gram-positive and gram-negative bacteria

The highly hydrophobic surface of waterfowl feathers confers outstanding self-cleaning capacity, effectively blocking external liquid ingress and reducing microbial adhesion (as described in the preceding section). Building on this passive protection, we endowed PPT@PG-BG with an active antibacterial function to more effectively prevent infection. In view of the escalating challenge of antibiotic resistance [36], we incorporated the natural polyphenol tannic acid (TA) as a non-antibiotic, broad-spectrum antibacterial agent. Acting via a multi-target mechanism—disrupting bacterial membranes, chelating essential metal ions, and inhibiting key enzymatic activities—TA suppresses both Gram-negative and Gram-positive bacteria [37,38], thereby further enhancing the material's self-cleaning performance and clinical potential.

**Fig. 4:**
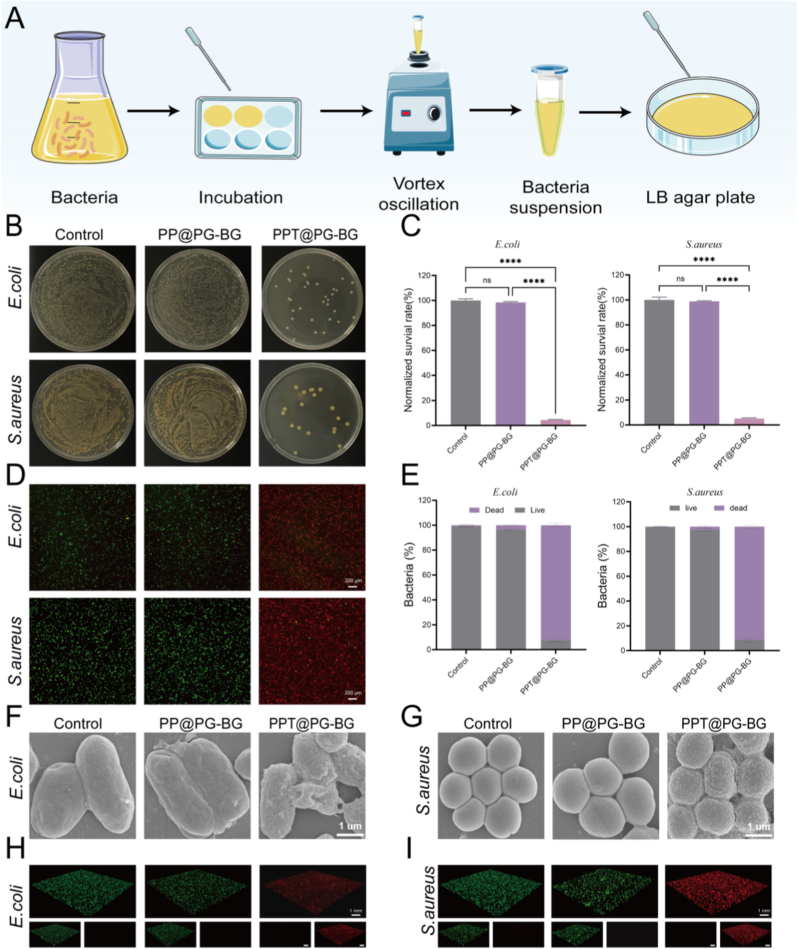
Evaluate the antibacterial performance of PPT@PG-BG. (A) Schematic illustration of the antibacterial assay procedures; (B) plate culture results of PPT@PG-BG materials after co-incubation with *Staphylococcus aureus* and *Escherichia coli*; (C) quantitative analysis of colony-forming units (CFU) from plate counting experiments; (D) fluorescence microscopy images of bacterial live/dead staining; (E) quantitative analysis of live/dead staining results; (F–G) SEM images of the morphological features of *E. coli* and *S. aureus* on the material surface; (H–I) 3D reconstruction visualizations of the survival status of *S. aureus* and *E. coli*, where live bacteria are shown in green and dead bacteria in red. (∗∗∗∗*p* < 0.0001 ns, no significant difference).

We systematically evaluated the antibacterial performance of PPT@PG-BG against *Escherichia coli* and *Staphylococcus aureus* using quantitative assays and morphological characterization. Plate counting (Fig. 4B and C) showed that tannic acid (TA)-loaded PPT@PG–BG markedly reduced colony survival to 4.41 % and 5.13 %, respectively. Live/dead staining (Fig. 4D and E) further revealed predominant green fluorescence (viable bacteria) in the TA-free PP@PG-BG group, whereas the TA-loaded PPT@PG-BG group exhibited abundant red fluorescence; quantitative analysis yielded survival rates of 7.73 % and 8.46 % for *E. coli* and *S. aureus*, in agreement with the plate results. Scanning electron microscopy (Fig. 4F and G) showed intact, smooth membranes in the control group, while bacteria exposed to PPT@PG-BG displayed wrinkling, collapse, and rupture—hallmarks of severe envelope damage—implicating membrane disruption as a key bactericidal pathway [39]. Studies have shown, as a natural polyphenol, TA acts via multiple targets: it forms hydrogen-bonding and hydrophobic interactions with cell-wall proteins and lipopolysaccharides, increasing membrane permeability and triggering leakage of intracellular contents [40,41]; additionally, it chelates essential metal ions (e.g., Fe^2+^, Mg^2+^), thereby inhibiting metal-dependent enzymatic activities and perturbing DNA replication and energy metabolism [42]. Together, these effects suppress both Gram-negative and Gram-positive bacteria. In sum, incorporation of TA endows PPT@PG-BG with non-antibiotic, broad-spectrum antibacterial activity, reduces bacterial adhesion and colonization, and synergizes with the material's self-cleaning properties to enhance its clinical anti-infective potential.

### 3.5 Bioactive ions released from the PPT@PG-BG membrane synergistically modulate the wound microenvironment

Diabetic wounds, as a paradigm of refractory chronic injuries, are subject to multiple pathological disturbances during the healing process, including persistent inflammatory responses, impaired angiogenesis, and elevated oxidative stress [43]. These factors collectively hinder tissue repair and cause the wound to remain in a prolonged inflammatory phase, severely compromising therapeutic outcomes. Conventional dressings, lacking the capacity for multi-targeted regulation, often do not address such a complex microenvironment, highlighting the urgent need for novel therapeutic systems capable of multidimensional intervention [44]. To this end, building upon the antibacterial and protective functions of the PPT@PG-BG material's outer layer, we further employed a multi-ion doping strategy to construct an “internal regulation” system centered on zinc (Zn^2+^), magnesium (Mg^2+^), and cerium (Ce^3+^). This synergistic approach aims to remodel the wound microenvironment and promote tissue regeneration. Specifically, zinc ions can markedly enhance fibroblast proliferation and modulate the expression of immune factors, thereby facilitating inflammation resolution and tissue reconstruction [45]. Magnesium ions improve local ischemia by promoting endothelial cell migration and angiogenesis [46]. Due to their exceptional antioxidant properties, Cerium ions efficiently scavenge excessive reactive oxygen species (ROS) and mitigate cellular oxidative damage [47]. The synergistic effects of these three functional ions enable multi-dimensional regulation of immune homeostasis, oxidative stress, and vascular regeneration, thereby substantially improving the overall efficiency of diabetic wound healing.

**Fig. 5:**
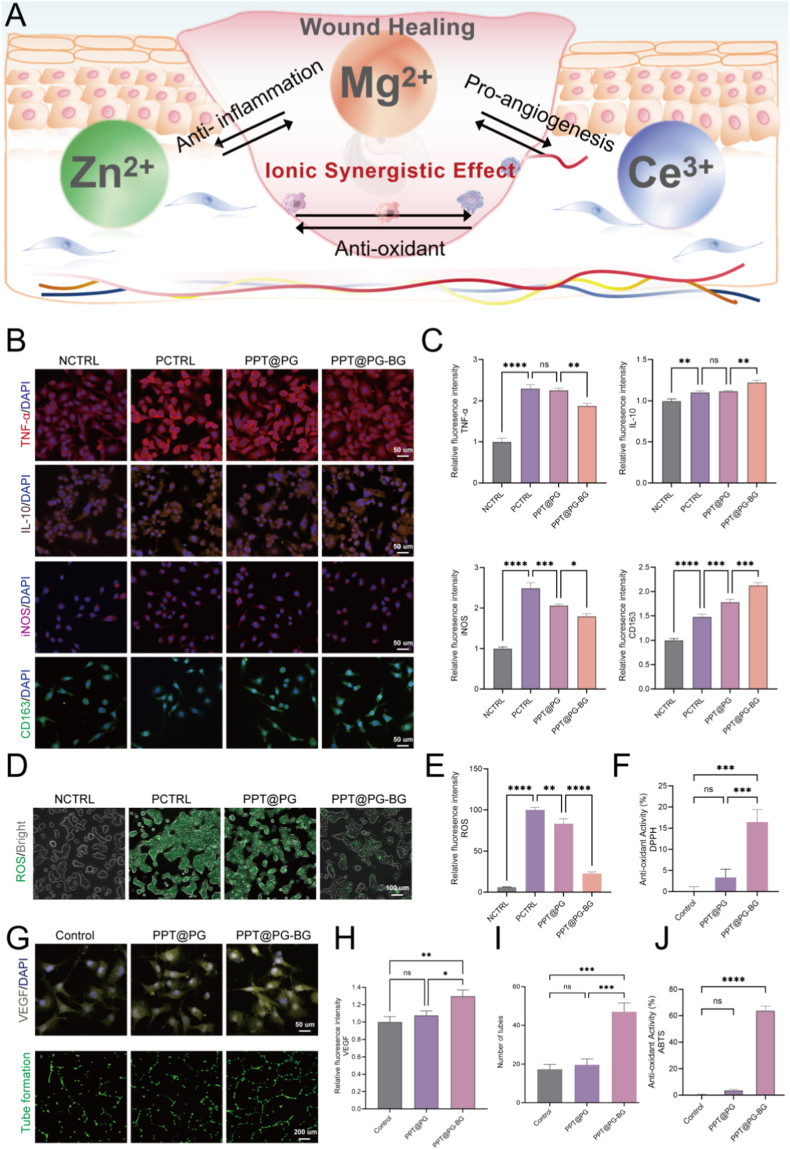
Ionic synergy modulates the local microenvironment and its biological effects. (A) Schematic of the ionic synergistic mechanism; (B) effects of ionic synergy on inflammatory markers TNF-α, IL-10, iNOS, and CD163; (C) quantitative analysis of inflammation-related indices; (D) fluorescence imaging of intracellular ROS; (E) quantification of ROS levels; (F) DPPH radical-scavenging capacity; (G) pro-angiogenic fluorescence imaging (VEGF expression and in vitro tube formation); (H–I) quantitative analysis of angiogenesis-related indices; (J) ABTS radical-scavenging capacity. (∗*p* < 0.05, ∗∗*p* < 0.01, ∗∗∗*p* < 0.001, ∗∗∗∗*p* < 0.0001; ns, no significant difference).

This study aims to systematically evaluate the mechanisms by which electrospun membranes loaded with bioactive glass (BG) regulate inflammatory responses, provide antioxidant protection, and promote angiogenesis during key wound healing processes. Initially, we investigated the effects of the materials on macrophage polarization by co-culturing them with RAW264.7 macrophages. As shown in Fig. 5B, the PPT@PG-BG group exhibited a marked reduction in the expression of the M1 macrophage marker iNOS and a significant decrease in the pro-inflammatory cytokine TNF-α. Concurrently, there was a pronounced increase in the expression of the M2 macrophage marker CD163 and the anti-inflammatory cytokine IL-10, with statistically significant differences compared to the PPT@PG group and the positive control group (*P* <∗∗0.01). Quantitative PCR results (Fig. S5) further corroborated these findings, demonstrating that the PPT@PG-BG electrospun membranes effectively promote the phenotypic shift of macrophages from a pro-inflammatory M1 state toward an anti-inflammatory M2 state. This transition significantly attenuates the inflammatory response and creates a more favorable environment for wound healing [48].

Furthermore, antioxidant activity is equally critical in diabetic wound healing, as it alleviates oxidative stress-induced cellular and tissue damage by neutralizing excessive reactive oxygen species (ROS) [49]. In this study, We assessed the radical-scavenging capacity of PPT@PG-BG in vitro using DPPH and ABTS assays (Fig. 5F and J). The PPT@PG group without bioactive glass (BG) exhibited minimal baseline antioxidant activity, with DPPH and ABTS scavenging rates of only 3.34 % and 3.60 %, respectively. By contrast, incorporation of BG markedly enhanced performance, with PPT@PG-BG achieving scavenging rates of 16.44 % and 63.76 %. In cellular experiments using ROS staining of HaCaT cells, the positive control group exhibited widespread green fluorescence compared to the negative control group, confirming successful model establishment (Fig. 5D). Notably, the PPT@PG-BG group demonstrated a marked reduction in both the intensity and area of green fluorescence relative to the PPT@PG group, with quantitative analysis (Fig. 5E) revealing a highly significant difference in the PPT@PG 1.5 %BG group (*P* < 0.001). These results indicate that the BG-loaded electrospun membranes possess superior antioxidant capacity, effectively attenuate oxidative stress-induced cellular injury, and indirectly modulate inflammation associated with oxidative stress.

Finally, inadequate blood supply is a significant barrier to diabetic wound healing, while angiogenesis is a cornerstone for effective tissue repair [50,51]. In this study, we further evaluated the pro-angiogenic potential of BG-loaded electrospun membranes. Co-culture of their extracts with human umbilical vein endothelial cells (HUVECs) revealed that the PPT@PG-BG group exhibited a clear advantage in angiogenesis assays (Fig. 5G). The total tube length and the number of branching points in the PPT@PG-BG group were significantly greater than those in the PPT@PG group without BG and the control group. Quantitative analysis of tube formation (Fig. 5I) further confirmed the significant enhancement in tube number in the PPT@PG-BG group (*P* < 0.001). Moreover, VEGF immunofluorescence staining (Fig. 5G)and quantitative PCR (Fig. S5c) demonstrated that VEGF expression was markedly upregulated in the PPT@PG-BG group. These findings indicate that BG-loaded electrospun membranes significantly promote angiogenesis by upregulating VEGF expression, providing a robust blood supply to the wound and accelerating the healing process.

In summary, the bioactive glass electrospun membranes loaded with magnesium, zinc, and cerium ions significantly accelerate diabetic wound healing through a Williams-like synergistic mechanism. Within this coordinated system, cerium ions attenuate oxidative stress by reducing reactive oxygen species (ROS) levels, suppressing excessive inflammatory responses, and preventing the onset of chronic inflammation, thus creating a favorable microenvironment for tissue repair. Simultaneously, cerium ions protect cell membranes and proteins from oxidative damage, preserve endothelial cell integrity, and promote the formation and stabilization of new blood vessels. Zinc ions play a pivotal role in immunomodulation by decreasing pro-inflammatory cytokine expression, enhancing the secretion of anti-inflammatory factors, and inducing the polarization of M1 macrophages toward the M2 phenotype. M2 macrophages, in turn, further upregulate angiogenic factors, facilitating neovascularization [51]. Magnesium ions enhance angiogenic potential by promoting cell migration and adhesion [52]. Moreover, robust vascularization provides the physiological foundation for effective antioxidant defense and inflammation resolution, ensuring uninterrupted wound healing. Through the integrated actions of cerium, zinc, and magnesium ions, the pathological cycle of “hyperosmolarity–hypoxia–chronic inflammation” characteristic of diabetic wounds can be effectively disrupted, enabling timely and demand-oriented tissue regeneration. This comprehensive multi-ion regulatory strategy offers a promising new paradigm for treating diabetic wounds.

### 3.6 The PPT@PG-BG membrane significantly accelerates cutaneous wound healing in a diabetic mouse model

The preceding investigations systematically validated the comprehensive advantages of the Janus-structured electrospun membrane in terms of material architecture, biocompatibility, outer-layer antibacterial barrier function, and inner-layer microenvironment modulation, highlighting its potential application for overcoming multiple obstacles in diabetic wound management. Building on these findings, this study further employed a diabetic-infected wound mouse model to evaluate the real-world therapeutic performance of this multifunctional dressing rigorously. All animal experiments were approved by the Ethics Committee of Nanfang Hospital, Southern Medical University (Ethics Approval Number: IACUC-LAC-20250416-005). Systematic in vivo experiments were conducted to verify its integrated efficacy in infection control, tissue repair, and functional regeneration under complex physiological conditions.

**Fig. 6:**
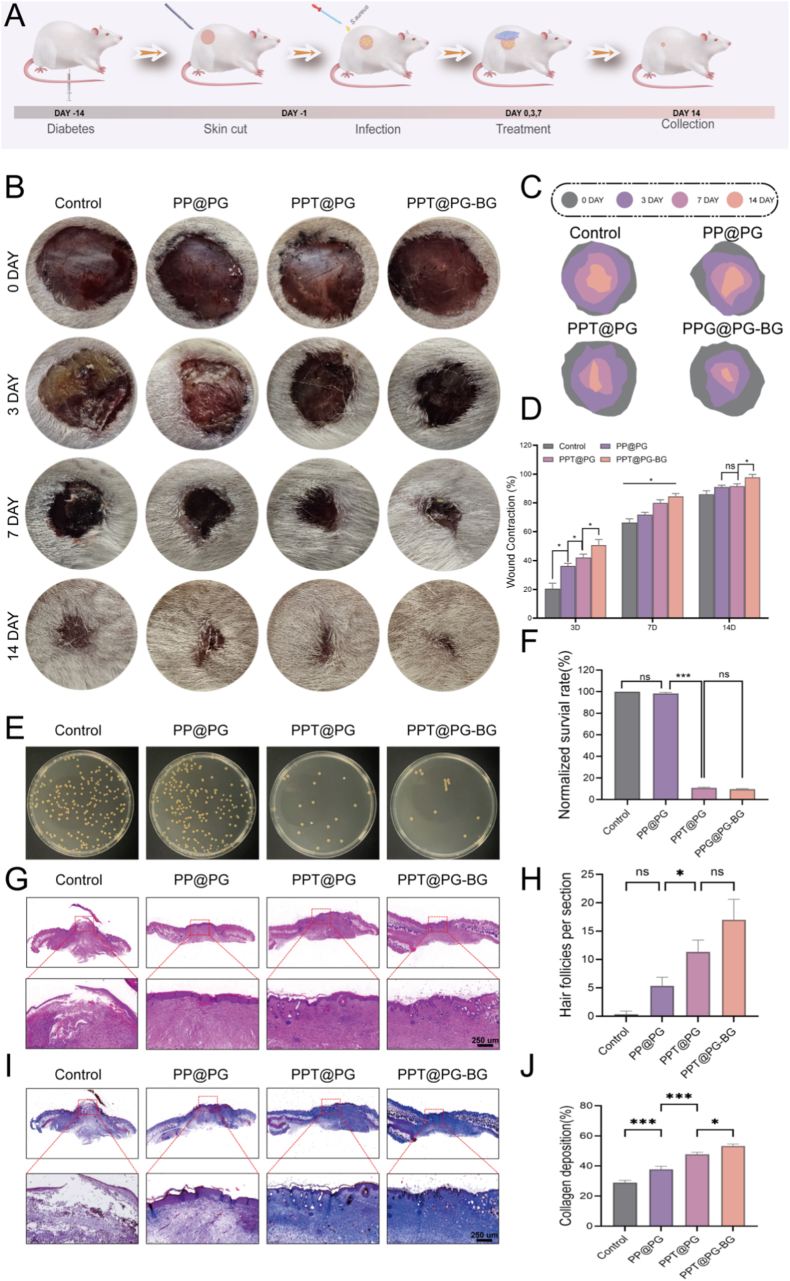
Macroscopic observations and histopathological analysis of animal experiments. (A) Schematic representation of the overall animal experimental workflow; (B) Macroscopic images of wound healing at different time points for each experimental group; (C) Visualization of wound closure areas at each time point; (D) Quantitative analysis of wound healing area over time; (E) Representative images of wound surface bacterial cultures on agar plates; (F) Quantitative statistics of bacterial colony numbers from plate cultures; (G) H&E staining images of wound tissues for assessment of inflammation and structural changes; (H) Quantitative analysis of hair follicle regeneration in H&E-stained sections; (I) Masson's trichrome staining images of wound tissues for evaluation of collagen deposition; (J) Quantification of collagen index in Masson-stained images (PP@PG refers to the bilayer electrospun membrane without tannic acid and bioactive glass; PPT@PG refers to the bilayer electrospun membrane without bioactive glass; ∗*p* < 0.05, ∗∗*p* < 0.01, ∗∗∗*p* < 0.001, ∗∗∗∗*p* < 0.0001, ns, no significant difference).

Following the successful establishment of the diabetic-infected wound mouse model wound healing in each experimental group was systematically evaluated in vivo. The wound sites were photographed on days 0, 3, 7, and 14 post-surgery to dynamically monitor the healing process at a macroscopic level (Fig. 6B). The results demonstrated a progressive reduction in wound area across all groups over time; however, notable differences were observed in healing rates and inflammatory responses. On day 3, the Control group and the PP@PG group, which lacked tannic acid, exhibited varying degrees of wound suppuration, indicating insufficient infection control. In contrast, neither the PPT@PG nor the PPT@PG-BG groups showed significant inflammatory reactions, underscoring their superior efficacy in managing infection. To further assess the in vivo antibacterial efficacy of the materials, wound exudates were collected on day 3 post-surgery for plate culturing (Fig. 6E). Extensive colonies of *S. aureus* and a few contaminant microorganisms were observed in the Control and PP@PG groups.

In contrast, the PPT@PG and PPT@PG-BG groups exhibited a marked reduction in bacterial colonies. Quantitative analysis revealed that the colony survival rates in the PPT@PG and PPT@PG-BG groups were 10.7 % and 9.63 %, respectively, consistent with the previously observed in vitro antibacterial results. These findings further confirm that tannic acid endows the material with excellent antibacterial properties. Quantitative analysis of wound contraction (Fig. 6C and D) revealed that the PPT@PG-BG group achieved a remarkable wound closure rate of 97.85 % by day 14, significantly surpassing all other groups and demonstrating exceptional capacity for promoting tissue regeneration. Histological evaluation of healed tissues using hematoxylin-eosin (HE) and Masson's trichrome staining further supported these findings. The PPT@PG-BG group (Fig. 6G) exhibited well-restored epidermal and dermal structures, orderly cellular arrangement, densely organized fibrous tissue, and a notable increase in the regeneration of skin appendages such as hair follicles [36]. Given the pivotal role of collagen in skin remodeling, collagen deposition was further assessed (Fig. 6I). Results showed that the PPT@PG-BG group possessed thicker, more orderly collagen fibers and a significantly higher level of collagen deposition. These results indicate that the BG-loaded PPT@PG-BG dressing enhances both epidermal and dermal regeneration, optimizes collagen fiber reconstruction, and promotes the recovery of skin appendages, thereby accelerating the healing of diabetic-infected wounds. In conclusion, the Janus dressing co-loaded with bioactive glass and tannic acid demonstrates clear advantages in infection control, tissue regeneration, and structural remodeling, markedly expediting the healing process of diabetic-infected wounds.

**Fig. 7:**
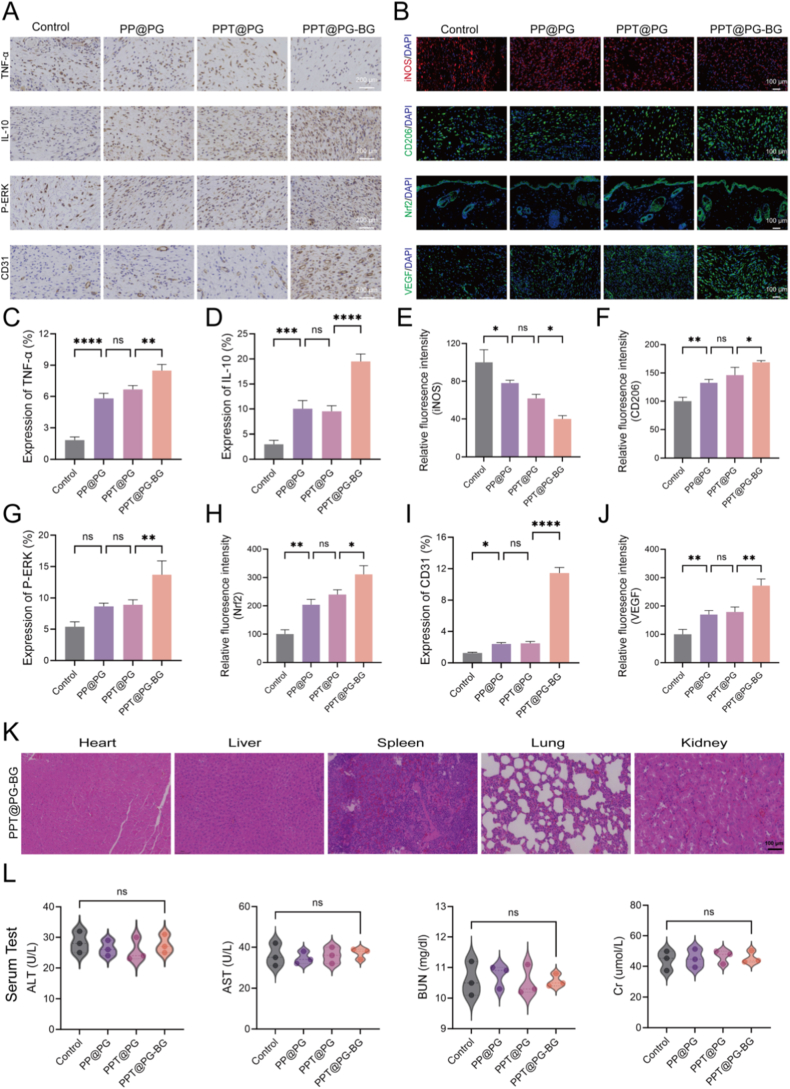
Immunohistochemical and immunofluorescent analysis of wound tissues. (A) Immunohistochemical staining of wound tissues for TNF-α, IL-10, p-ERK, and CD31; (B). Immunofluorescent staining of wound tissues for iNOS, CD206, Nrf2, and VEGF; (C). Quantitative analysis of TNF-α expression; (D). Quantitative analysis of IL-10 expression; (E). Quantitative analysis of iNOS expression; (F). Quantitative analysis of CD206 expression; (G). Quantitative analysis of p-ERK expression; (H). Quantitative analysis of Nrf2 expression; (I). Quantitative analysis of CD31 expression; (J). Quantitative analysis of VEGF expression; (K). Histopathological sections of major organs at day 14 post-operation; (L). Serum biochemical analysis at day 7 post-operation. (∗*p* < 0.05, ∗∗*p* < 0.01, ∗∗∗*p* < 0.001, ∗∗∗∗*p* < 0.0001, ns, no significant difference).

To further elucidate the mechanisms by which the bilayer electrospun membrane promotes diabetic wound healing, immunofluorescence, and immunohistochemical analyses were performed on mouse wound tissues. Under diabetic conditions, metabolic dysregulation induces persistent oxidative stress, damaging endothelial cells, disrupting angiogenic signaling pathways, and exacerbating inflammation and extracellular matrix (ECM) disorganization. Previous in vitro studies have confirmed that this bilayer dressing, which has synergistic ionic effects, possesses excellent antioxidant properties. At the in vivo level, the PPT@PG-BG group exhibited markedly increased expression of phosphorylated ERK (p-ERK) and the key antioxidant transcription factor Nrf2 in wound tissues (Fig. 7A and B). Notably, p-ERK functions as a critical node in regulating oxidative stress pathways, modulating the balance between cell survival and apoptosis, while nuclear translocation of Nrf2 activates the transcription of a battery of antioxidant-related genes [53]. These findings suggest that the PPT@PG-BG material enhances the tissue's antioxidant response by boosting p-ERK signaling and Nrf2 pathway activation, thereby mitigating oxidative damage. Regarding inflammation regulation, the PPT@PG-BG group exhibited a significant reduction in the expression of the pro-inflammatory cytokine TNF-α and a marked upregulation of the anti-inflammatory cytokine IL-10 (Fig. 7A and B), indicating strong anti-inflammatory potential. Macrophage polarization was assessed to clarify further the effects of the dressing on the immune microenvironment. Immunofluorescence results demonstrated a pronounced decrease in iNOS expression (an M1 phenotype marker) and a substantial increase in CD206 expression (an M2 phenotype marker) in the PPT@PG-BG group, suggesting that the material facilitates the transition of macrophages from a pro-inflammatory M1 phenotype to a pro-regenerative M2 phenotype. This polarization reprogramming is conducive to attenuating local inflammation and enhancing tissue repair capacity. Moreover, the bilayer dressing exhibited pronounced pro-angiogenic effects. Immunostaining revealed a significant elevation of the vascular endothelial marker CD31 in the PPT@PG-BG group (Fig. 7A), indicative of active neovascularization. Further analysis with VEGF staining demonstrated a marked upregulation of this group's vascular endothelial growth factor (VEGF) expression, providing molecular evidence for the enhanced angiogenic response. The concurrent upregulation of VEGF and CD31 suggests that the material promotes the reconstruction of blood supply in diabetic wounds by orchestrating immune and angiogenic signaling pathways [26,54]. In addition, serum biochemistry and primary organ histopathological analyses were performed to assess the in vivo biocompatibility of the bilayer electrospun membrane. The results showed no statistically significant differences in measured indices between the bilayer membrane and control groups (Fig. 7K and L, and Fig. S6). Collectively, these findings validate the efficacy and safety of the PPT@PG-BG bilayer electrospun membrane in vivo.

These findings suggest that the Janus bilayer dressing loaded with bioactive glass and tannic acid (PPT@PG-BG) may accelerate tissue regeneration by orchestrating multiple levels of wound microenvironmental regulation. Specifically, the dressing appears to synergistically activate the p-ERK/Nrf2 antioxidant pathway, reshape the inflammatory cytokine profile by suppressing TNF-α and upregulating IL-10, induce macrophage polarization from the pro-inflammatory M1 phenotype toward the pro-regenerative M2 phenotype, and promote neovascularization via the VEGF/CD31 signaling axis. Through these concerted effects-mitigating oxidative stress, remodeling the immune microenvironment, and restoring blood supply, dressing effectively reconstructs the diabetic wound healing microenvironment and expedites tissue regeneration.

**Fig. 8:**
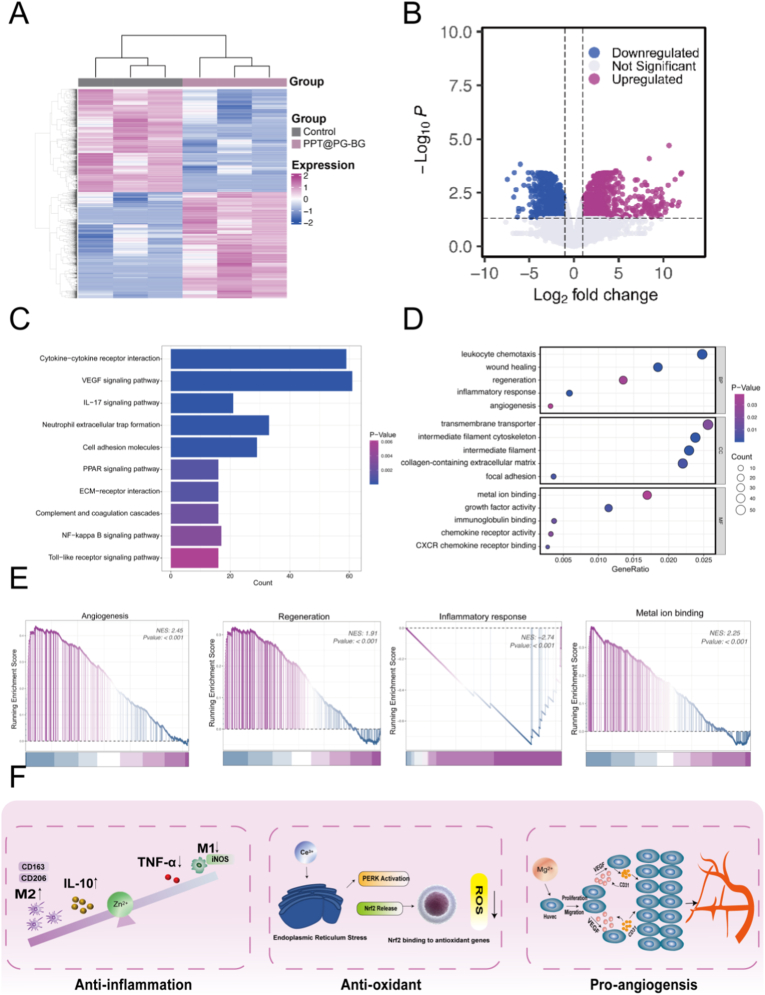
Transcriptomic analysis and mechanistic insights following PPT@PG-BG dressing intervention in diabetic wound tissues. (A). Clustered heatmap of differentially expressed genes (DEGs); (B). Volcano plot illustrating the number and significance distribution of up- and downregulated genes (selection criteria: |log_2_FC| > 1, *p* < 0.05); (C). Gene Ontology (GO) enrichment analysis shows significant enrichment of upregulated genes in key biological processes such as leukocyte chemotaxis, tissue repair, regenerative inflammation regulation, and angiogenesis; (D). Kyoto Encyclopedia of Genes and Genomes (KEGG) pathway enrichment analysis; (E). Gene Set Enrichment Analysis (GSEA) further demonstrates that the dressing dynamically coordinates the activation of signaling pathways related to angiogenesis, epithelial regeneration, inflammatory response, and metal ion metabolism. (F)-schematic diagram of the proposed underlying mechanisms.

To further elucidate the molecular mechanisms underlying the accelerated in vivo healing of diabetic infected wounds by PPT@PG-BG, transcriptome sequencing analysis was performed in this study. Wound tissue samples from the control and PPT@PG-BG groups (n = 3 per group) were subjected to RNA sequencing. Differentially expressed genes (DEGs) were visualized using heatmaps (Fig. 8A) and volcano plots (Fig. 8B). The results revealed that compared to the control group, 2030 genes were significantly upregulated, and 2038 genes were significantly downregulated in the PPT@PG-BG-treated group, indicating that the intervention elicited extensive molecular modulation at the transcriptional level. Gene Ontology (GO) enrichment analysis showed that upregulated genes were predominantly associated with key biological processes such as leukocyte chemotaxis, wound healing, regulation of regenerative inflammation, and angiogenesis (Fig. 8C), all closely related to tissue regeneration. Further KEGG pathway enrichment analysis revealed that PPT@PG-BG markedly inhibited the activity of the IL-17 and NF-κB signaling pathways, thereby reducing the expression of canonical pro-inflammatory cytokines such as TNF-α and IL-1β and reinforcing its role in negative regulation of inflammation. Concurrently, the material significantly activated the VEGF signaling pathway, PPAR pathway, and cell adhesion molecule (CAM) pathways, collectively promoting endothelial cell proliferation, epithelial cell migration, and neovascularization to accelerate tissue repair. Gene set enrichment analysis (GSEA) further demonstrated that angiogenesis-related pathways were rapidly activated during the early stage of wound healing. In contrast, pathways associated with tissue proliferation and repair were progressively upregulated in the mid-to-late stages, reflecting the robust and dynamic regulatory capability of PPT@PG-BG throughout different phases of repair. Notably, the sustained release of multiple metal ions (Mg^2+^, Zn^2+^, Ce^3+^) from PPT@PG-BG synergistically activated metal ion metabolism-related pathways, with this ionic modulation contributing to antioxidant defense, inflammation suppression, and angiogenesis.

In summary, the Janus bilayer dressing incorporating tannic acid and multifunctional bioactive glass orchestrates multiple regenerative mechanisms-including the regulation of oxidative stress, inflammatory responses, and angiogenesis-to remodel the repair microenvironment of diabetic infected wounds at the molecular, cellular, and tissue levels. This dressing activates the p-ERK/Nrf2 antioxidant pathway, induces macrophage polarization toward the M2 phenotype, and suppresses pro-inflammatory pathways such as NF-κB and IL-17. Simultaneously, it enhances VEGF/CD31-mediated angiogenesis, while the synergistic effects of released metal ions further augment its immunomodulatory and regenerative capacity. Collectively, these concerted actions markedly improve infection control, tissue reconstruction, and functional recovery, highlighting this strategy's considerable potential for treating complex diabetic ulcers.

### 3.7 Discussion and limitations

We have successfully engineered a novel PPT@PG-BG Janus nanofibrous membrane via electrospinning. This membrane integrates asymmetric wettability, multi-ion synergistic therapy, and excellent biocompatibility, demonstrating significant potential to accelerate wound healing in both in vitro and in vivo experiments. Its core advantage lies in simulating a natural strategy of “external defense and internal regulation.” The hydrophobic outer layer, loaded with tannic acid (PPT), provides a physical barrier and resists external infection through a coordinated “active + passive” mechanism. Concurrently, the hydrophilic inner layer actively and synergistically modulates the complex wound microenvironment by controllably releasing bioactive ions such as Mg^2+^, Zn^2+^, and Ce^3+^. This process effectively suppresses inflammation, promotes angiogenesis, and collaboratively enhances tissue repair. Such a design, which integrates physical functionality with active biotherapy, offers a highly promising new paradigm for the treatment of recalcitrant wounds like diabetic foot ulcers.

However, to translate this technology from the laboratory to the clinic, we must prudently consider both its potential and its limitations. First, the Janus dressing designed in this study holds considerable potential for clinical translation. The electrospinning process is amenable to large-scale, low-cost production, which is a critical prerequisite for commercialization [55]. Furthermore, the dressing's design aligns with clinical handling practices, making it easy to apply and replace. Its superior exudate management capability is expected to reduce the frequency of dressing changes, thereby alleviating patient suffering and nursing burden. Second, long-term biosafety and performance stability are crucial areas for future investigation. Although this study confirmed its good biocompatibility in a two-week animal model, its long-term in vivo degradation products and potential systemic effects require more in-depth research, especially for chronic wounds with longer healing cycles [56]. Future work should involve larger-scale, longer-term animal studies (e.g., in rabbit or porcine models) for a more comprehensive evaluation. Additionally, testing the performance stability of the dressing in an environment that simulates authentic wound fluid is necessary. Finally, regarding risk assessment and control, the primary potential risk originates from the dosage of bioactive ions. While the ion concentrations used in our study demonstrated a good safety profile, individual metabolic capacities for metal ions may vary, particularly in patients with conditions like renal insufficiency. Therefore, defining a “therapeutic window” that is both safe and effective for a broad population is essential. Moreover, as with any implantable material, a theoretical risk of inducing a foreign body response or allergic reaction remains.

Nevertheless, these potential risks are relatively manageable when weighed against the significant clinical benefits this technology could offer. For patients suffering from diabetic foot ulcers and facing the risk of amputation, an advanced dressing that can simultaneously address the four core challenges—exudate management, infection control, inflammation, and angiogenesis—holds immense clinical value. It has the potential to significantly increase healing rates, shorten treatment duration, reduce healthcare costs, and thereby dramatically improve patient quality of life.

In summary, although the path from bench to bedside is challenging, the Janus dressing we propose, which integrates physical regulation with active biotherapy, has demonstrated enormous potential as a next-generation smart wound management system. Future work will focus on addressing the aforementioned translational challenges to ultimately realize its clinical value.

## 4 Conclusion

Inspired by the asymmetric wetting architecture of waterfowl feathers, this study developed a biomimetic Janus nanofiber dressing with dual functions of infection barrier and microenvironment modulation, presenting an integrated “outer protection + inner regeneration” strategy for wound management. The outer layer, composed of PLA and tannic acid, exhibits excellent hydrophobicity, self-cleaning properties, and intrinsic antibacterial activity, effectively preventing pathogen invasion and fluid infiltration while providing a sustained anti-infection barrier. The inner layer, fabricated by electrospinning bioactive glass, enables dynamic modulation of the local wound microenvironment. Both in vitro and in vivo experiments demonstrated that the dressing, through the synergistic action of multiple ions, activates the p-ERK/Nrf2 antioxidant pathway, promotes macrophage polarization towards the M2 phenotype, and enhances VEGF/CD31-mediated angiogenesis. These effects notably alleviate the pathological state of “hyperosmolarity–hypoxia–chronic inflammation” in wounds, reconstructing the immune-regeneration axis. Furthermore, transcriptomic and tissue-level analyses revealed marked enrichment and regulatory potential in key biological pathways involved in neovascularization, epithelial regeneration, and inflammation modulation. In summary, the plant polyphenol-ion–ion synergistic Janus dressing constructed in this study not only achieves effective antimicrobial defense through interface engineering but also systematically remodels the diabetic wound healing microenvironment via intrinsic multi-ion bioactivity, demonstrating broad application prospects for the treatment of complex infected chronic wounds and providing new insights for the development of advanced functional wound dressings.

## CRediT authorship contribution statement

**Chaoyang Huang:** Writing – original draft, Formal analysis, Data curation, Conceptualization. **Lianglong Chen:** Writing – original draft, Formal analysis, Data curation, Conceptualization. **Huihui Zhang:** Writing – original draft, Formal analysis, Data curation, Conceptualization. **Bo Liu:** Resources, Project administration, Methodology, Investigation. **Hai Zhou:** Resources, Project administration, Methodology, Investigation. **Yanqi Chen:** Validation, Supervision, Software. **Xian Li:** Validation, Supervision, Software. **Xiaoyang Liu:** Validation, Supervision, Software. **Limin Zhao:** Visualization, Validation, Supervision, Software. **Xue Wang:** Validation, Supervision, Software. **Min Wu:** Validation, Supervision, Software. **Shuaijie Li:** Validation, Supervision, Software. **Dan Yi:** Validation, Supervision, Software. **Chunyu Liu:** Writing – review & editing, Visualization, Funding acquisition. **Haobo Pan:** Writing – review & editing, Visualization, Funding acquisition. **Lei Yang:** Writing – review & editing, Visualization, Funding acquisition.

## Declaration of competing interest

We declare that no conflict of interest exits in the submission of this manuscript, and manuscript is approved by all authors for publication. I would like to declare on behalf of my co-authors that the work described was original research that has not been published previously, and not under consideration for publication elsewhere, in whole or in part. All the authors listed have approved the manuscript that is enclosed.

## Data Availability

Data will be made available on request.
