# Supplementary material for: Feather-inspired janus interfaces with spatiotemporal ionic programming for diabetic wound infection control and regenerative healing
Source: Mater Today Bio. 2025 Nov 1;35:102506. doi: 10.1016/j.mtbio.2025.102506 (PMC12630035; doi:10.1016/j.mtbio.2025.102506)
Supplement: Multimedia component 1 [file mmc1.docx]

**Supplementary Materials**

**Feather-Inspired Janus Interfaces with Spatiotemporal Ionic Programming for Diabetic Wound Infection Control and Regenerative Healing**

**Authors:** Chaoyang Huang^a#^, Lianglong Chen^a#^, Huihui Zhang^a#^, Bo Liu^c^, Hai Zhou^d^, Yanqi Chen^a^, Xian Li^a^, Xiaoyang Liu^a^, Limin Zhao^b,e^, Xue Wang^f^, Min Wu^a^, Shuaijie Li^b^, Dan Yi^b^, Chunyu Liu^b,f^*, Haobo Pan^b,f^*, Lei Yang^a^*

**Affiliations:**

^a^Department of Burns, Nanfang Hospital, Southern Medical University, Jingxi Street, Baiyun District, Guangdong, 510515, People's Republic of China.

^b^Shenzhen Institutes of Advanced Technology, Chinese Academy of Sciences, ShenzhenGuangdong, 518055, China

^c^Department of Burns and Plastic Surgery, Liuzhou Worker’s Hospital, Fourth Affiliated Hospital of Guangxi Medical University, Liuzhou, Guangxi, 545000, China.

^d^Yunfu People's Hospital, Central Laboratory of Yunfu People's Hospital, Yunfu City, 527399, PR China

^e^Geriatric Medicine Department, Shenzhen Longhua District Central Hospital, Shenzhen, 518000, PR China

^f^Shenzhen Healthemes Biotechnology Co., Ltd, Shenzhen, 518102, P.R. China

**^#^These authors contributed equally to this work.**

***Corresponding author:**

Lei Yang: yuanyang@smu.edu.cn

Haobo Pan: [hb.pan@siat.ac.cn](mailto:Hb.pan@siat.ac.cn)

Chunyu Liu: [cy.liu@siat.ac.cn](mailto:cy.liu@siat.ac.cn)

**目录**

**[1. Characterization of bioactive glass. 3](#_Toc1303)**

**[2. Characterization of the outer electrospun layer. 4](#_Toc2984)**

**[3. Antibacterial properties and microstructure of the electrospun outer layer 6](#_Toc32004)**

**[4. Hydrophilicity and hydrophobicity of the electrospun inner layer. 8](#_Toc15603)**

**[5. qRT-PCR 9](#_Toc6030)**

**[6. In vivo toxicity assessment 10](#_Toc30006)**

**[7. Mechanical Properties and Ion Release Profiles of Different Electrospun Membranes 11](#_Toc7617)**

1. **Characterization of bioactive glass.**


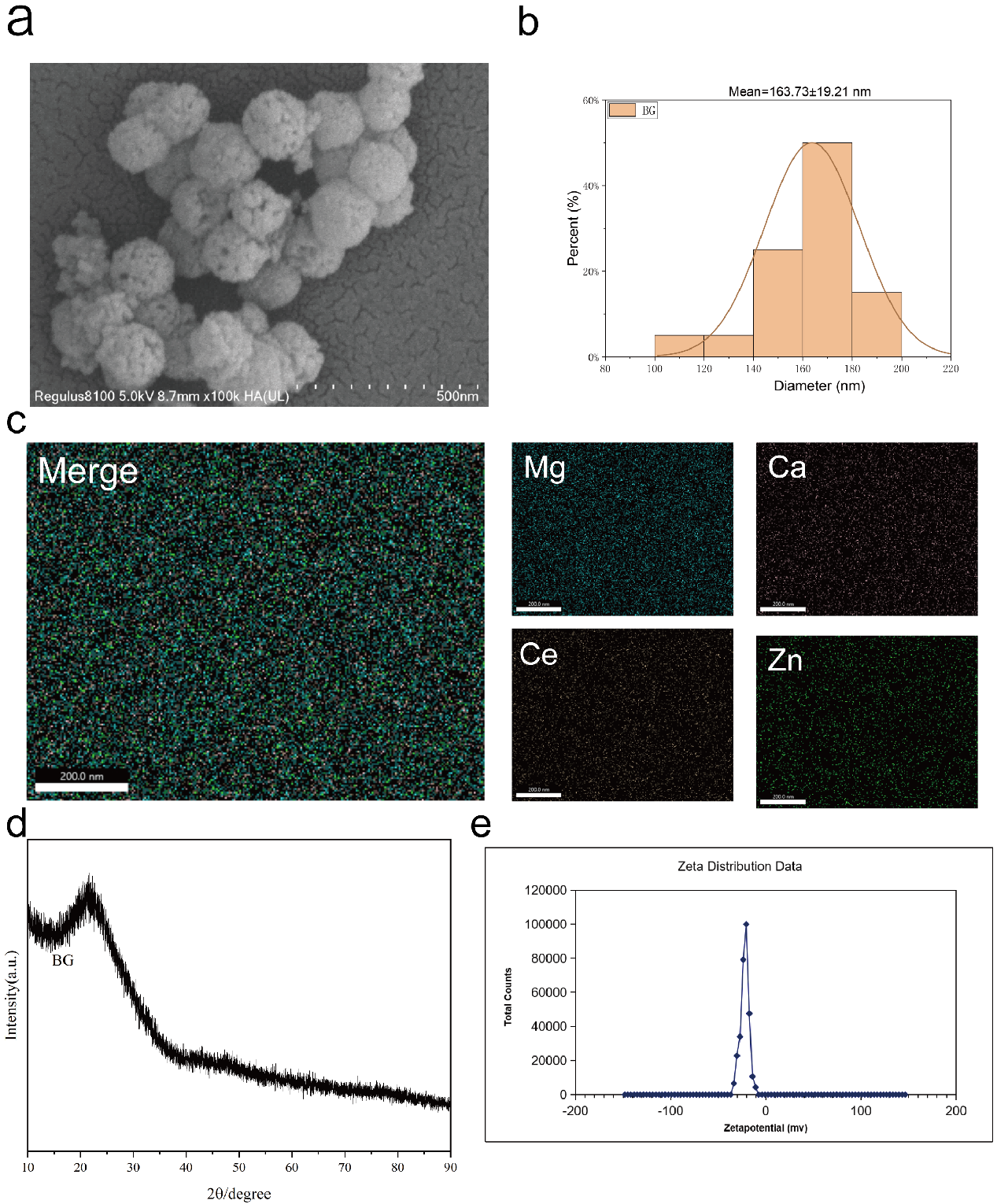


**Fig. S1.** (a) Scanning electron microscopy (SEM) image of bioactive glass; (b) Particle size distribution of bioactive glass; (c) Energy-dispersive spectroscopy (EDS) elemental mapping of bioactive glass; (d) X-ray diffraction (XRD) pattern of bioactive glass; (e) Zeta potential of bioactive glass.

1. **Characterization of the outer electrospun layer.**


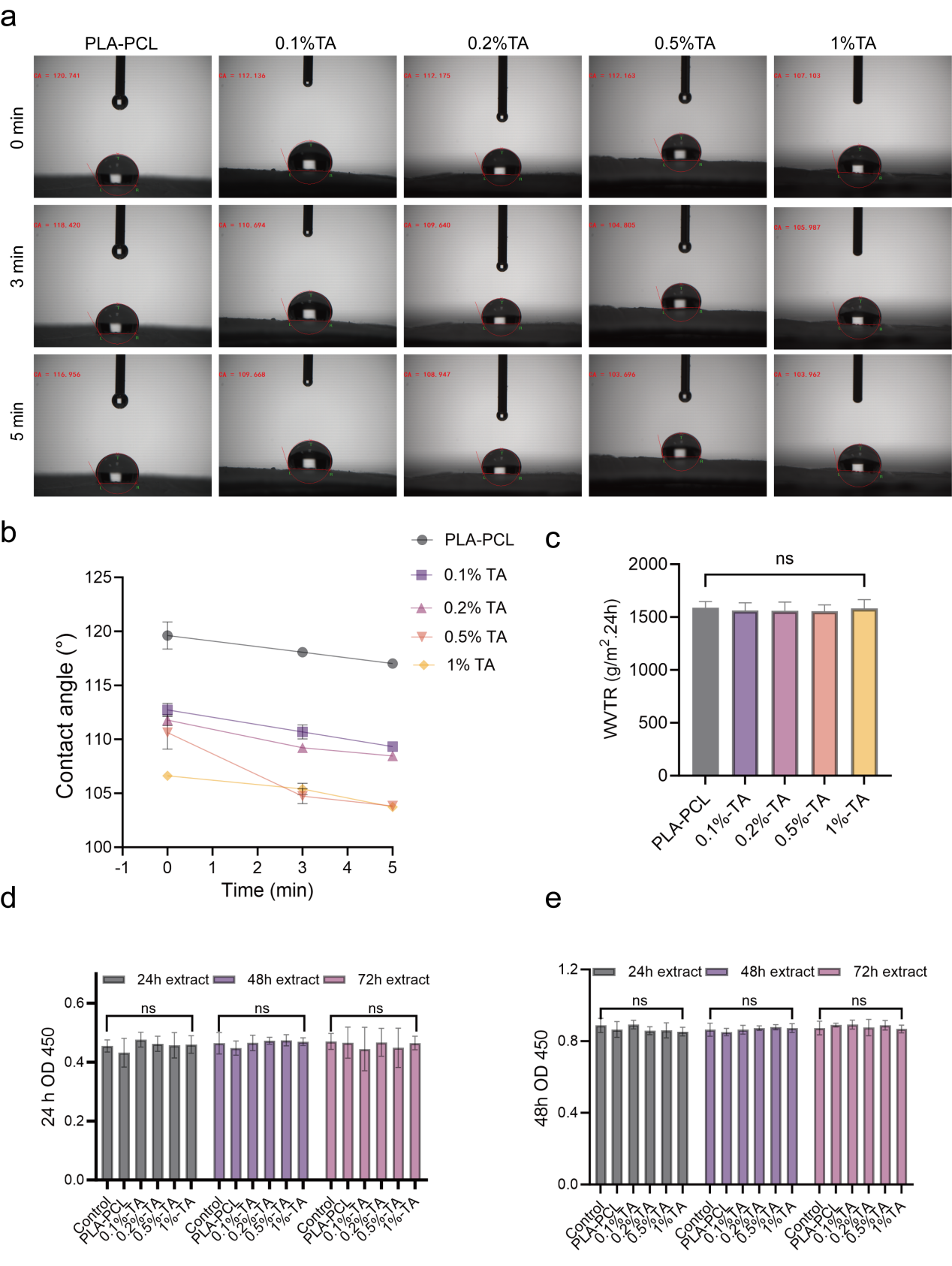


**Fig. S2.** **Characterization of the outer electrospun layer.** (a) Water contact angle measurements of the outer electrospun layer loaded with varying concentrations of tannic acid; (b) Quantitative analysis of water contact angles; (c) Water vapor transmission rate (WVTR) test results of the materials; (d-e) CCK-8 assay results for cell viability of L929 cells co-cultured with extracts of the outer electrospun samples for 24 and 48 hours.

1. **Antibacterial properties and microstructure of the electrospun outer layer**


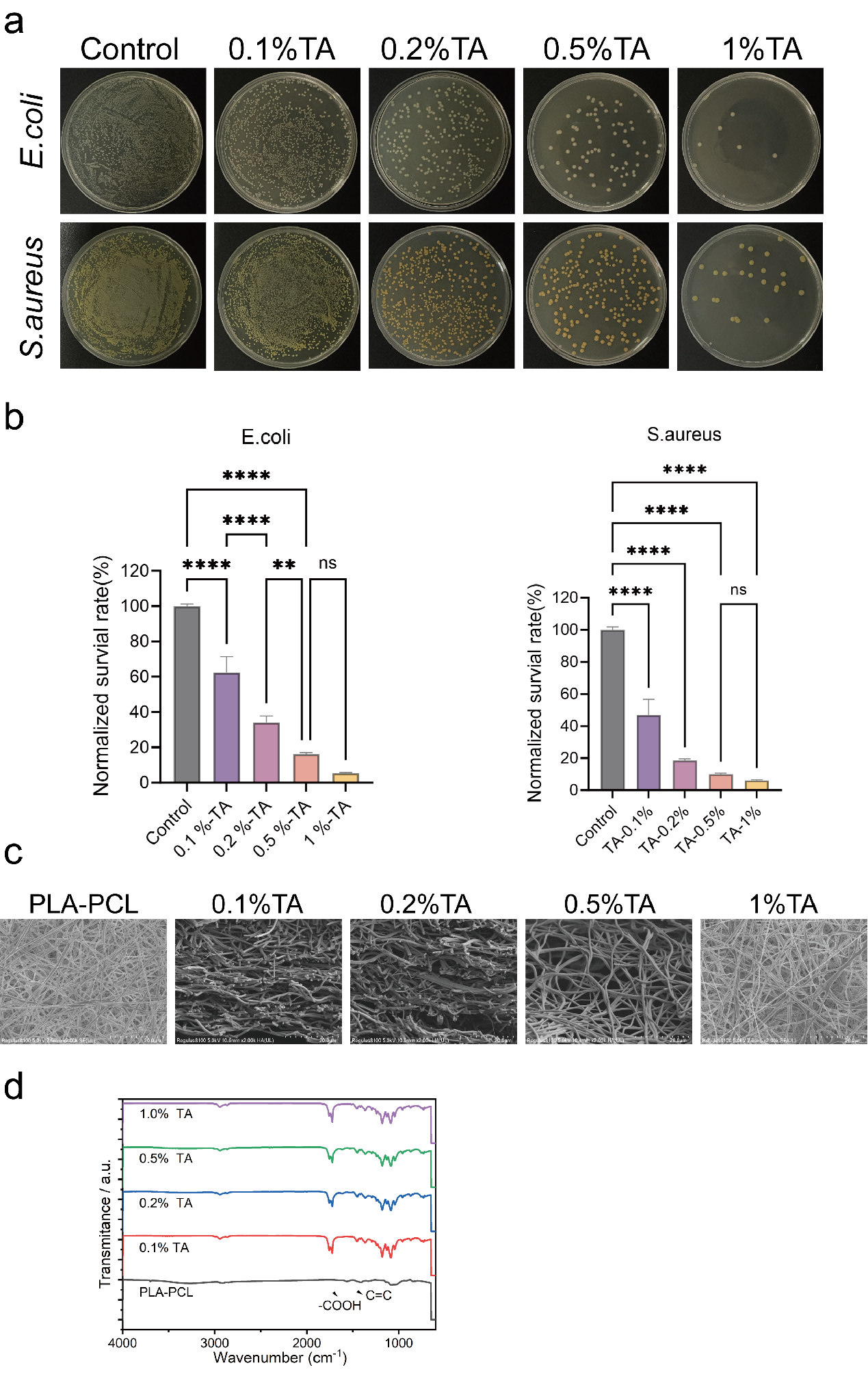


**Fig. S3.** (a) Bacterial colony images of outer electrospun layers loaded with different concentrations of tannic acid; (b) Quantitative analysis of antibacterial rates of the outer electrospun layer with varying tannic acid concentrations against Escherichia coli and Staphylococcus aureus; (c) Scanning electron microscopy (SEM) image of the outer electrospun layer; (d) Fourier transform infrared (FTIR) spectra of the outer electrospun layer loaded with different concentrations of tannic acid.

**4. Hydrophilicity and hydrophobicity of the electrospun inner layer.**


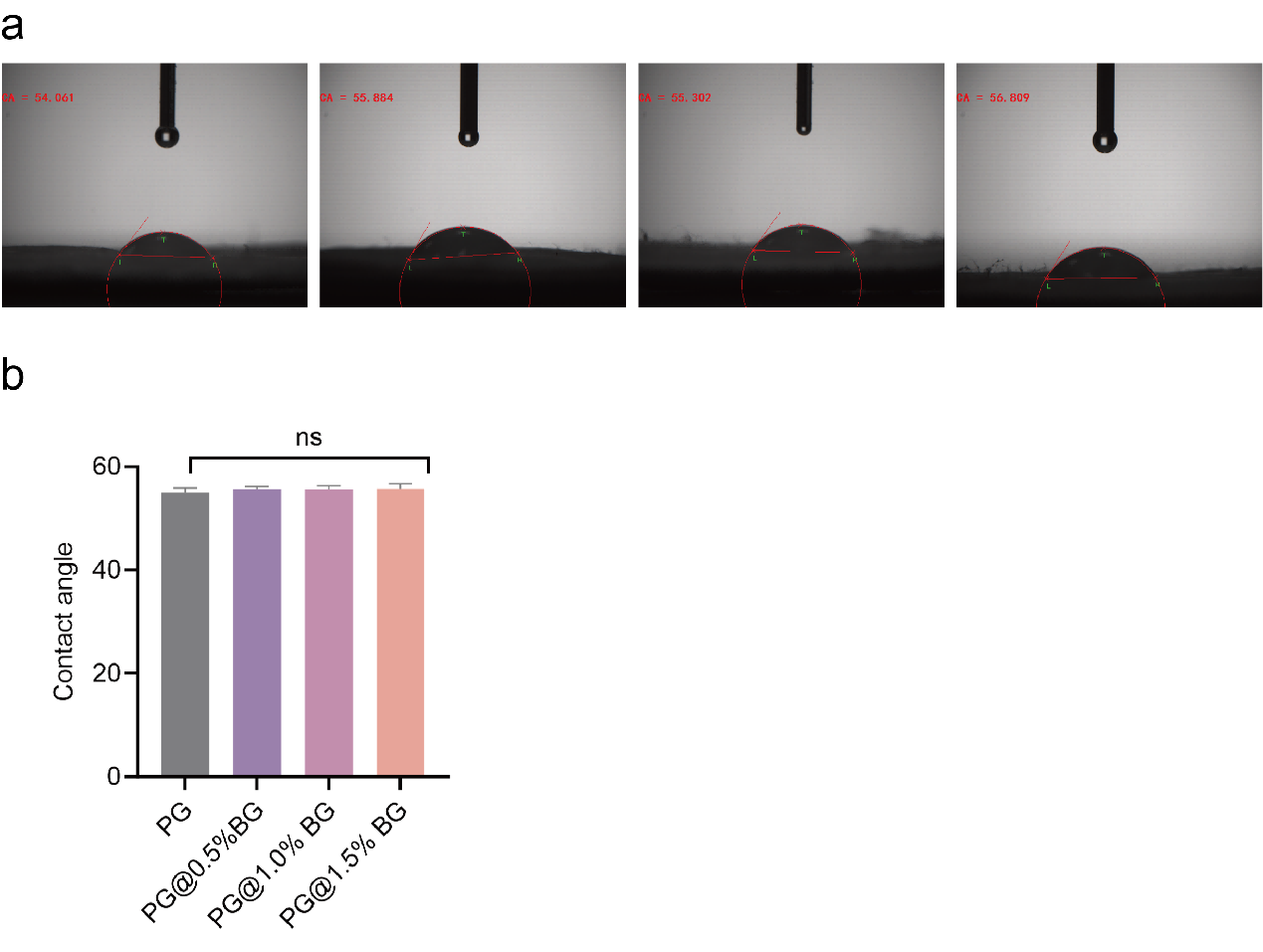


**Fig. S4.** (a) Water contact angle of the inner layer of the bilayer electrospun membrane; (b) Quantitative analysis of water contact angles.

**5. qRT-PCR**


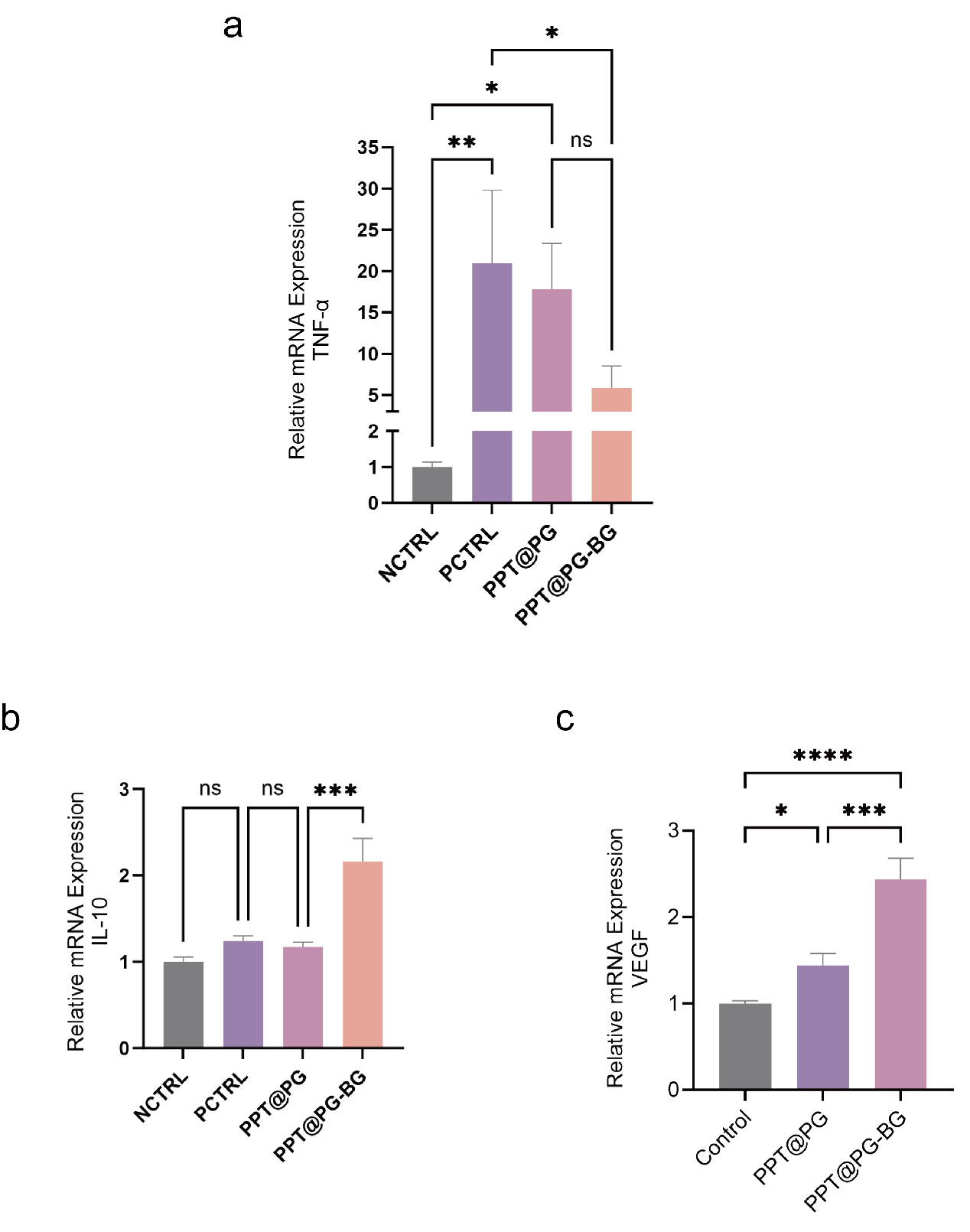


**Fig. S5. qRT-PCR** (a) Quantitative analysis of TNF-α expression by qPCR; (b) Quantitative analysis of IL-10 expression by qPCR; (c) Quantitative analysis of VEGF expression by qPCR.

**6. In vivo toxicity assessment**


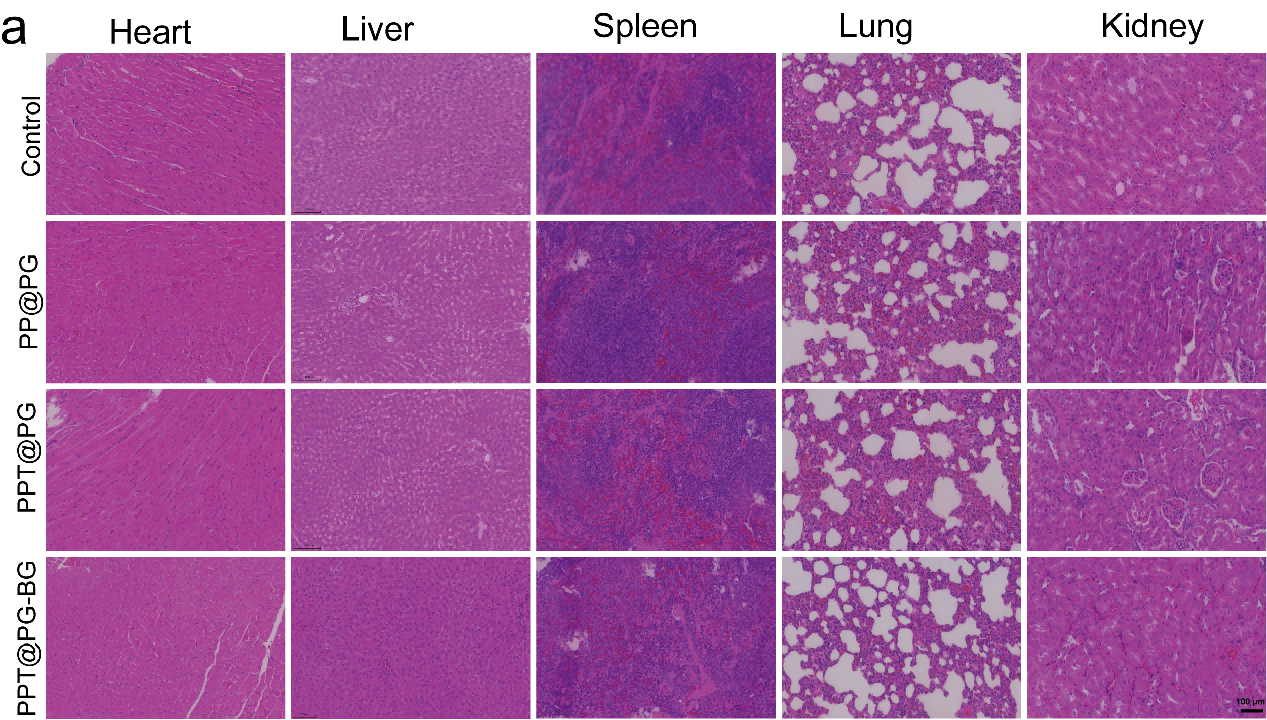


**Fig. S6.** H&E staining images of mouse organs (heart, liver, spleen, lungs and kidneys) after 14 days in different treatment groups.

1. **Mechanical Properties and Ion Release Profiles of Different Electrospun Membranes**

**Fig. S7** (a-c) Ion release curves (including Mg^2+^, Zn^2+^, and Ce^3+^ ions) from membranes loaded with different concentrations of BG; (d) Stress-strain curves of various electrospun membranes.
